# Supplementary material for: Use and misuse of temperature normalisation in meta-analyses of thermal responses of biological traits
Source: PeerJ. 2018 Feb 9;6:e4363. doi: 10.7717/peerj.4363 (PMC5808315; doi:10.7717/peerj.4363)
Supplement: Appendix S1 [file peerj-06-4363-s001.pdf]

Appendix A from D. G. Kontopoulos et al., "Use and misuse of temperature normalisation in meta-analyses of thermal responses of biological traits"

Supplementary material, mathematical derivations, and data sources

Contents

A1 Reference temperature values used in the recent literature 2

A2 Mathematical derivations 3
- A2.1 The importance of normalisation of B_0 at an explicit T_{ref} value 3
- A2.2 Derivation of the T_{pk} parameter 3
- A2.3 Derivation of the P_{pk} parameter 4

A3 The conditional inference tree 4
- A3.1 Description of the algorithm 4
- A3.2 Evaluation of its performance 5

A4 Definition of the probability density function of the beta distribution 5

A5 List of studies whose data were used in this paper 6

List of Tables

A1 Table of specified T_{ref} values in some studies that employed the Sharpe-Schoolfield model. . . . 2

A2 The confusion matrix from the data-driven conditional inference tree. 5

A1 Reference temperature values used in the recent literature

Table A1: Table of specified T_{ref} values in some studies that employed the Sharpe-Schoolfield model.

Study	T_{ref} (°C)
Ungerer, M. J., M. P. Ayres, and M. J. Lombardero. 1999. Climate and the northern distribution limits of *Dendroctonus frontalis* Zimmermann (Coleoptera: Scolytidae). Journal of Biogeography 26:1133–1145.	24.85
Wohlfahrt, G., M. Bahn, E. Haubner, I. Horak, W. Michaeler, K. Rottmar, U. Tappeiner, and A. Cernusca. 1999. Inter-specific variation of the biochemical limitation to photosynthesis and related leaf traits of 30 species from mountain grassland ecosystems under different land use. Plant, Cell & Environment 22:1281–1296.	20.01
Hopp, M. J., and J. A. Foley. 2001. Global-scale relationships between climate and the dengue fever vector, *Aedes aegypti*. Climatic Change 48:441–463.	24.85
Depinay, J.-M. O., C. M. Mbogo, G. Killeen, B. Knols, J. Beier, J. Carlson, J. Dushoff, P. Billingsley, H. Mwambi, J. Githure, et al. 2004. A simulation model of African *Anopheles* ecology and population dynamics for the analysis of malaria transmission. Malaria Journal 3:1.	24.85
Barmak, D. H., C. O. Dorso, M. Otero, and H. G. Solari. 2014. Modelling interventions during a dengue outbreak. Epidemiology and Infection 142:545–561.	24.85
Barneche, D. R., M. Kulbicki, S. R. Floeter, A. M. Friedlander, J. Maina, and A. P. Allen. 2014. Scaling metabolism from individuals to reef-fish communities at broad spatial scales. Ecology Letters 17:1067–1076.	20
Fand, B. B., H. E. Z. Tonnang, M. Kumar, A. L. Kamble, and S. K. Bal. 2014. A temperature-based phenology model for predicting development, survival and population growth potential of the mealybug, *Phenacoccus solenopsis* Tinsley (Hemiptera: Pseudococcidae). Crop Protection 55:98–108.	16.88, 25.01, 33.86, 34.47 *
Nealis, V. G., and J. Régnière. 2014. An individual-based phenology model for western spruce budworm (Lepidoptera: Tortricidae). The Canadian Entomologist 146:306–320.	24.85
Kang, S. H., J.-H. Lee, and D.-S. Kim. 2015. Temperature-dependent fecundity of overwintered *Scirtothrips dorsalis* (Thysanoptera: Thripidae) and its oviposition model with field validation. Pest Management Science 71:1441–1451.	25
Simoy, M. I., M. V. Simoy, and G. A. Canziani. 2015. The effect of temperature on the population dynamics of *Aedes aegypti*. Ecological Modelling 314:100–110.	24.85
Barneche, D., M. Kulbicki, S. Floeter, A. Friedlander, and A. Allen. 2016. Energetic and ecological constraints on population density of reef fishes. Proceedings of the Royal Society of London. Series B, Biological sciences 283:20152186.	20
Padfield, D., G. Yvon-Durocher, A. Buckling, S. Jennings, and G. Yvon-Durocher. 2016. Rapid evolution of metabolic traits explains thermal adaptation in phytoplankton. Ecology Letters 19:133–142.	25

* Note: *T_{ref} in Fand et al. 2014 was treated as a model parameter and not as a constant, allowing its value to vary across fits.*

Figure A1: Bar plot of T_{ref} values in table A1, rounded to the nearest integer for simplicity. A clear bias towards $T_{\mathrm{ref}} \approx 25$°C emerges, probably due to the use of this value in the original paper by Schoolfield et al. (1981).

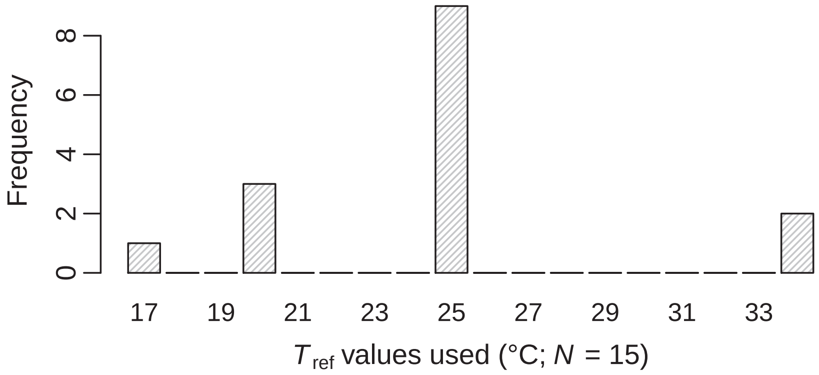

A2 Mathematical derivations

A2.1 The importance of normalisation of B_0 at an explicit T_{ref} value

The Sharpe-Schoolfield equation (eq. (1)) can be reformulated without T_{ref}, i.e. without temperature normalisation of the B_0 parameter, by changing the numerator from $e^{\frac{-E}{k} \cdot \left(\frac{1}{T} - \frac{1}{T_{\mathrm{ref}}}\right)}$ to $e^{\frac{-E}{k} \cdot \left(\frac{1}{T}\right)}$. This implies that:

$$\frac{1}{T_{\mathrm{ref}}} \approx 0 \implies T_{\mathrm{ref}} \approx \infty$$

Therefore, removing the T_{ref} value from the Sharpe-Schoolfield equation leads to B_0 being estimated at an extremely high – and biologically meaningless – temperature.

A2.2 Derivation of the T_{pk} parameter

To obtain the equation of T_{pk}, the temperature at which trait performance peaks, we first differentiate the Sharpe-Schoolfield equation (eq. (1)) with respect to T, using the quotient rule:

$$\frac{\mathrm{d}}{\mathrm{d}T} \frac{B_0 \cdot e^{\frac{-E}{k} \cdot \left(\frac{1}{T} - \frac{1}{T_{\mathrm{ref}}}\right)}}{1 + e^{\frac{E_{\mathrm{D}}}{k} \cdot \left(\frac{1}{T_{\mathrm{h}}} - \frac{1}{T}\right)}} =$$

$$= \frac{\frac{B_0 \cdot E}{k \cdot T^2} \cdot e^{\frac{-E}{k} \cdot \left(\frac{1}{T} - \frac{1}{T_{\mathrm{ref}}}\right)} \cdot \left(1 + e^{\frac{E_{\mathrm{D}}}{k} \cdot \left(\frac{1}{T_{\mathrm{h}}} - \frac{1}{T}\right)}\right) - \frac{B_0 \cdot E_{\mathrm{D}}}{k \cdot T^2} \cdot e^{\frac{E_{\mathrm{D}}}{k} \cdot \left(\frac{1}{T_{\mathrm{h}}} - \frac{1}{T}\right)} \cdot e^{\frac{-E}{k} \cdot \left(\frac{1}{T} - \frac{1}{T_{\mathrm{ref}}}\right)}}{\left(1 + e^{\frac{E_{\mathrm{D}}}{k} \cdot \left(\frac{1}{T_{\mathrm{h}}} - \frac{1}{T}\right)}\right)^2}$$

We then solve for T, by setting the above quantity equal to zero. This will give the temperature at which the rate of change of the trait with respect to temperature is zero (T_{pk}). As the denominator and $\frac{B_0}{k \cdot T^2}$ will always be positive, the remaining part of the numerator has to be equal to zero:

$$E \cdot e^{\frac{-E}{k}\cdot\left(\frac{1}{T}-\frac{1}{T_{\mathrm{ref}}}\right)} + E \cdot e^{\frac{-E}{k}\cdot\left(\frac{1}{T}-\frac{1}{T_{\mathrm{ref}}}\right)+\frac{E_{\mathrm{D}}}{k}\cdot\left(\frac{1}{T_{\mathrm{h}}}-\frac{1}{T}\right)} = E_{\mathrm{D}} \cdot e^{\frac{E_{\mathrm{D}}}{k}\cdot\left(\frac{1}{T_{\mathrm{h}}}-\frac{1}{T}\right)-\frac{E}{k}\cdot\left(\frac{1}{T}-\frac{1}{T_{\mathrm{ref}}}\right)} \implies$$

$$\implies E \cdot e^{\frac{-E}{k}\cdot\left(\frac{1}{T}-\frac{1}{T_{\mathrm{ref}}}\right)} = (E_{\mathrm{D}} - E) \cdot e^{\frac{E_{\mathrm{D}}}{k}\cdot\left(\frac{1}{T_{\mathrm{h}}}-\frac{1}{T}\right)-\frac{E}{k}\cdot\left(\frac{1}{T}-\frac{1}{T_{\mathrm{ref}}}\right)}$$

Taking the natural logarithm of both sides and solving for T leads to:

$$\ln\frac{E}{E_{\mathrm{D}} - E} = \frac{E_{\mathrm{D}}}{k} \cdot \left(\frac{1}{T_{\mathrm{h}}} - \frac{1}{T}\right) \implies T = \frac{-E_{\mathrm{D}} \cdot T_{\mathrm{h}}}{k \cdot T_{\mathrm{h}} \cdot \ln\frac{E}{E_{\mathrm{D}} - E} - E_{\mathrm{D}}} = T_{\mathrm{pk}}$$

A2.3 Derivation of the P_{pk} parameter

P_{pk} represents the peak of the thermal response curve (i.e. the maximum trait performance) and can be estimated by setting $T = T_{\mathrm{pk}}$ in the Sharpe-Schoolfield equation (eq. (1)):

$$P_{\mathrm{pk}} = B(T_{\mathrm{pk}}) = B_0 \cdot \frac{e^{\frac{-E}{k}\cdot\left(\frac{1}{T_{\mathrm{pk}}}-\frac{1}{T_{\mathrm{ref}}}\right)}}{1 + e^{\frac{E_{\mathrm{D}}}{k}\cdot\left(\frac{1}{T_{\mathrm{h}}}-\frac{1}{T_{\mathrm{pk}}}\right)}}$$

A3 The conditional inference tree

A3.1 Description of the algorithm

Conditional inference trees attempt to classify a response variable based on combinations of other predictor variables. This is done through a tree-like structure (see fig. 3) where terminal nodes (or leaves) are the predicted classifications, whereas all other nodes up to the root of the tree correspond to predictor variables. Essentially, each internal node is a decision point in the model at which two alternative paths can be followed, depending on the value of the corresponding predictor. The topology of the tree is constructed through a statistical significance approach, consisting of non-parametric tests for each particular node, corrected for multiple testing to avoid overfitting. As a result, the predictors are unbiased and the model does not require cross-validation, often performed in other classes of models to assess overfitting. Overall, conditional inference trees allow identification of complicated nonlinear associations between a response variable and its predictors, which are otherwise quite difficult to distinguish.

A3.2 Evaluation of its performance

Table A2: The confusion matrix from the data-driven conditional inference tree.

		True condition			
		Training dataset		Testing dataset	
		above	below	above	below
Predicted condition	above	**135**	4	**20**	5
	below	8	**1611**	3	**377**

This table allows for assessing the performance of a classifying model, both against the data that were used to train it and against new data (testing dataset). The number of cases for which the model predictions are in agreement with the true condition of the data are shown in bold. Based on these results, we can deduce that the model is both sensitive and specific, able to recognize most combinations of parameters that result in B_0 being above (true positive rate in the training/testing datasets: 94.41% and 86.96%) or below P_{pk} (true negative rate in the training/testing datasets: 99.75% and 98.69%).

A4 Definition of the probability density function of the beta distribution

The probability density function of the beta distribution for $x \in [0, 1]$, and α, $\beta > 0$ is

$$f(x|\alpha, \beta) = \frac{x^{\alpha-1} \cdot (1-x)^{\beta-1}}{\displaystyle\int_0^1 x^{\alpha-1} \cdot (1-x)^{\beta-1} \mathrm{d}x}.$$

Figure A2: Probability density functions of the beta distribution for different values of the α and β parameters.

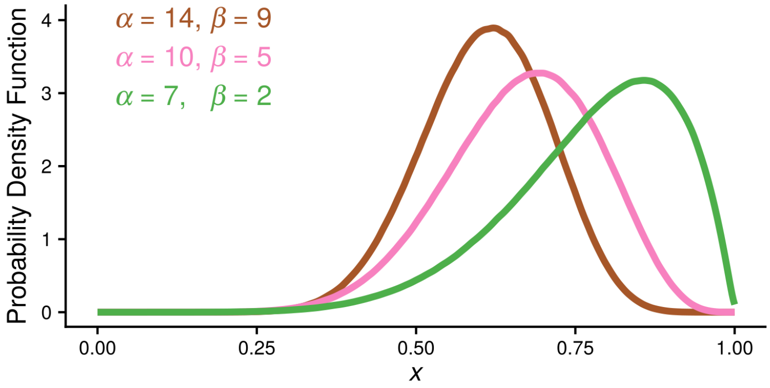

A5 List of studies whose data were used in this paper

Abrini, J., H. Naveau, and E.-J. Nyns. 1994. Clostridium autoethanogenum, sp. nov., an anaerobic bacterium that produces ethanol from carbon monoxide. Archives of Microbiology 161:345–351.

Adamec, L. 1997. Photosynthetic characteristics of the aquatic carnivorous plant Aldrovanda vesiculosa. Aquatic Botany 59:297–306.

Adams, M. S. 1970. Adaptations of Aplectrum hyemale to the Environment: Effects of Preconditioning Temperature on Net Photosynthesis. Bulletin of the Torrey Botanical Club 97:219.

Adams, M. S., and M. M. Fayyaz. 1979. Temperature acclimation of net photosynthesis in relation to growth of a cold hardy Chrysanthemum. Oecologia 39:239–247.

Admiraal, W. 1976. Influence of light and temperature on the growth rate of estuarine benthic diatoms in culture. Marine Biology 39:1–9.

Aelion, C., and S. Chisholm. 1985. Effect of temperature on growth and ingestion rates of Favella sp. Journal of plankton research 7:821–830.

Aghdam, H. R., Y. Fathipour, G. Radjabi, and M. Rezapanah. 2009. Temperature-dependent development and temperature thresholds of codling moth (Lepidoptera: Tortricidae) in Iran. Environmental entomology 38:885–895.

Ahlgren, G. 1978. Growth of Oscillatoria agardhii in chemostat culture. 2. Dependence of growth constants on temperature. *In* Symposium: Experimental Use of Algal Cultures in Limnology 26-28 October 1976, Sandefjord, Norway. Internationale Vereinigung fur Theoretische und Angewandte Limnologie, Mittelungen. 21.

———. 1987. Temperature functions in biology and their application to algal growth constants. Oikos pages 177–190.

Albrizio, R., and P. Steduto. 2003. Photosynthesis, respiration and conservative carbon use efficiency of four field grown crops. Agricultural and Forest Meteorology 116:19–36.

Ali, R. M. 1970. The influence of suspension density and temperature on the filtration rate of Hiatella arctica. Marine Biology 6:291–302.

Anderson, M. T., J. M. Kiesecker, D. P. Chivers, and A. R. Blaustein. 2001. The direct and indirect effects of temperature on a predator prey relationship. Canadian Journal of Zoology 79:1834–1841.

Angilletta, M. J., T. Hill, and M. A. Robson. 2002. Is physiological performance optimized by thermoregulatory behavior?: a case study of the eastern fence lizard, Sceloporus undulatus. Journal of Thermal Biology 27:199–204.

Arbab, A., D. C. Kontodimas, and M. R. McNeill. 2008. Modeling embryo development of Sitona discoideus Gyllenhal (Coleoptera: Curculionidae) under constant temperature. Environmental entomology 37:1381–1388.

Atkin, O., C. Holly, and M. Ball. 2000. Acclimation of snow gum (Eucalyptus pauciflora) leaf respiration to seasonal and diurnal variations in temperature: the importance of changes in the capacity and temperature sensitivity of respiration. Plant, Cell & Environment 23:15–26.

Atkin, O. K. 2000. Leaf Respiration of Snow Gum in the Light and Dark. Interactions between Temperature and Irradiance. PLANT PHYSIOLOGY 122:915–924.

ATKIN, O. K., I. SCHEURWATER, and T. L. PONS. 2006. High thermal acclimation potential of both photosynthesis and respiration in two lowland Plantago species in contrast to an alpine congeneric. Global Change Biol 12:500–515.

Autumn, K., R. B. Weinstein, and R. J. Full. 1994. Low cost of locomotion increases performance at low temperature in a nocturnal lizard. Physiological Zoology pages 238–262.

Azcon-Bieto, J., G. Farquhar, and A. Caballero. 1981. Effects of temperature, oxygen concentration, leaf age and seasonal variations on the CO2 compensation point of Lolium perenne L. Planta 152:497–504.

Azcón-Bieto, J., and C. B. Osmond. 1983. Relationship between Photosynthesis and Respiration The Effect of Carbohydrate Status on the Rate of CO2 Production by Respiration in Darkened and Illuminated Wheat Leaves. Plant Physiology 71:574–581.

Baars, J. 1981. Autecological investigations on marine diatoms. 2. Generation times of 50 species. Hydrobiological Bulletin 15:137–151.

———. 1985. Autecological investigations on marine diatoms. 4: Biddulphia aurita (Lyngb.) Brebisson et GodeyA succession of spring diatoms. Hydrobiological Bulletin 19:109–116.

Babel, S., S. Takizawa, and H. Ozaki. 2002. Factors affecting seasonal variation of membrane filtration resistance caused by Chlorella algae. Water Research 36:1193–1202.

Baddeley, M. S., B. Ferry, and E. J. Finegan. 1971. A new method of measuring lichen respiration: response of selected species to temperature, pH and sulphur dioxide. The Lichenologist 5:18–25.

Badger, M., O. Björkman, and P. Armond. 1982. An analysis of photosynthetic response and adaptation to temperature in higher plants: temperature acclimation in the desert evergreen Nerium oleander L. Plant, Cell & Environment 5:85–99.

Baek, S. H., S. Shimode, and T. Kikuchi. 2008. Growth of dinoflagellates, Ceratium furca and Ceratium fusus in Sagami Bay, Japan: The role of temperature, light intensity and photoperiod. Harmful Algae 7:163–173.

BAILEY, P. C. E. 1989. The effect of water temperature on the functional response of the water stick insect Ranatra dispar (Heteroptera: Nepidae). Austral Ecology 14:381–386.

Bailey, P. C. E. 2010. The Effect of Density and Temperature on the Swimming and Aggregating Behaviour of the Backswimmer, Anisops deanei (Heteroptera: Notonectidae) and Subsequent Encounter Rate with a Sit-and-Wait Predator. Ethology 77:44–57.

Baldwin, N. S. 1957. Food Consumption and Growth of Brook Trout at Different Temperatures. Transactions of the American Fisheries Society 86:323–328.

Baltz, D. M., P. B. Moyle, and N. J. Knight. 1982. Competitive Interactions Between Benthic Stream Fishes, Riffle Sculpin, Cottus gulosus , and Speckled Dace, Rhinichthys osculus . Can. J. Fish. Aquat. Sci. 39:1502–1511.

Band-Schmidt, C. J. 2004. Effects of growth medium, temperature, salinity and seawater source on the growth of Gymnodinium catenatum (Dinophyceae) from Bahia Concepcion, Gulf of California, Mexico. Journal of Plankton Research 26:1459–1470.

Barker, H. A. 1935. The culture and physiology of the marine dinoflagellates. Archives of Microbiology 6:157–181.

Barko, J. W., and R. M. Smart. 1981. Comparative influences of light and temperature on the growth and metabolism of selected submersed freshwater macrophytes. Ecological monographs pages 219–235.

Batty, R., and J. Blaxter. 1992. The effect of temperature on the burst swimming performance of fish larvae. Journal of Experimental Biology 170:187–201.

Batty, R., J. Blaxter, and K. Fretwell. 1993. Effect of temperature on the escape responses of larval herring, Clupea harengus. Marine Biology 115:523–528.

Bauwens, D., T. Garland, A. M. Castilla, and R. V. Damme. 1995. Evolution of Sprint Speed in Lacertid Lizards: Morphological, Physiological and Behavioral Covariation. Evolution 49:848.

Bell, E. C. 1993. Photosynthetic response to temperature and desiccation of the intertidal alga Mastocarpus papillatus. Marine Biology 117:337–346.

Benider, A., M. Tahiri, M. Belkoura, and A. Dauta. 2001. Interaction des facteurs héliothermiques sur la croissance de trois espèces du genre Scenedesmus. Pages 257–266 *in* Annales de Limnologie-International Journal of Limnology. Vol. 37. Cambridge Univ Press.

Bennett, A. F. 1980. The thermal dependence of lizard behaviour. Animal Behaviour 28:752–762.

Bergman, E. 1987. Temperature-dependent differences in foraging ability of two percids, Perca fluviatilis and Gymnocephalus cernuus. Environ Biol Fish 19:45–53.

Bergmann, P. 2006. Effects of temperature on maximum acceleration, deceleration and power output during vertical running in geckos. Journal of Experimental Biology 209:1404–1412.

Bernacchi, C. J., E. L. Singsaas, C. Pimentel, A. R. P. Jr, and S. P. Long. 2001. Improved temperature response functions for models of Rubisco-limited photosynthesis. Plant, Cell and Environment 24:253–259.

Binder, B. J., and D. M. Anderson. 2004. PHYSIOLOGICAL AND ENVIRONMENTAL CONTROL OF GERMINATION IN SCRIPPSIELLA TROCHOIDEA (DINOPHYCEAE) RESTING CYSTS1. Journal of Phycology 23:99–107.

Bjorkman, O., H. A. Mooney, and J. Ehleringer. 1975. Photosynthetic responses of plants from habitats with contrasting thermal environments. Carnegie Inst. Wash. Yearbook 74:743–748.

Blankley, W. F., and R. A. Lewin. 1976. Temperature responses of a coccolithophorid, Cricosphaera carterae, measured in a simple and inexpensive thermal-gradient device. Limnology and Oceanography 21:457–462.

Bollmann, J., C. Klaas, and L. E. Brand. 2010. Morphological and Physiological Characteristics of Gephyrocapsa oceanica var. typica Kamptner 1943 in Culture Experiments: Evidence for Genotypic Variability. Protist 161:78–90.

Bongi, G., and S. Long. 1987. Light-dependent damage to photosynthesis in olive leaves during chilling and high temperature stress. Plant, Cell & Environment 10:241–249.

Bouarab, L., M. Loudiki, and A. Dauta. 2002. Autotrophic and mixotrophic growth of microalgae Micractinium pusillum Fres. isolated from wastewater stabilization pond: influence of light and temperature. Revue des Sciences de l'Eau (France) .

Bouchamma, E. O., and M. Derraz. 2004. Interaction of light and temperature effects on the growth rate of three Cyanobacteria species isolated from El kansera impoundment (Morocco). Algological Studies 113:129–141.

Bouterfas, R., M. Belkoura, and A. Dauta. 2002. Light and temperature effects on the growth rate of three freshwater algae isolated from a eutrophic lake. Hydrobiologia 489:207–217.

Bowman, J. P., D. S. Nichols, and T. A. McMeekin. 1997. Psychrobacter glacincola sp. nov., a Halotolerant, Psychrophilic Bacterium Isolated from Antarctic Sea Ice. Systematic and Applied Microbiology 20:209–215.

Boyd, P. W., T. A. Rynearson, E. A. Armstrong, F. Fu, K. Hayashi, Z. Hu, D. A. Hutchins, R. M. Kudela, E. Litchman, M. R. Mulholland, et al. 2013. Marine phytoplankton temperature versus growth responses from polar to tropical waters–outcome of a scientific community-wide study. PLoS One 8:e63091.

Brand, L., and R. Guillard. 1981. The effects of continuous light and light intensity on the reproduction rates of twenty-two species of marine phytoplankton. Journal of Experimental Marine Biology and Ecology 50:119–132.

Bravo, I., and D. M. Anderson. 1994. The effects of temperature, growth medium and darkness on excystment and growth of the toxic dinoflagellate Gymnodinium catenatum from northwest Spain. Journal of Plankton Research 16:513–525.

Breeze, V., and J. Elston. 1978. Some effects of temperature and substrate content upon respiration and the carbon balance of field beans (Vicia faba L.). Annals of Botany 42:863–876.

Breitbarth, E., A. Oschlies, and J. LaRoche. 2007. Physiological constraints on the global distribution of Trichodesmium? effect of temperature on diazotrophy. Biogeosciences 4:53–61.

Brett, J. R. 1967. Swimming Performance of Sockeye Salmon (Oncorhynchus nerka) in relation to Fatigue Time and Temperature . J. Fish. Res. Bd. Can. 24:1731–1741.

Briand, J.-F., C. Leboulanger, J.-F. Humbert, C. Bernard, and P. Dufour. 2004. CYLINDROSPERMOPSIS RACIBORSKII (CYANOBACTERIA) INVASION AT MID-LATITUDES: SELECTION, WIDE PHYSIOLOGICAL TOLERANCE, OR GLOBAL WARMING? Journal of Phycology 40:231–238.

Brock, V., and L. Kofoed. 1987. Species specific irrigatory efficiency in Cardium (Cerastoderma) edule (L.) and C. lamarcki (Reeve) responding to different environmental temperatures. Biological oceanography 4:211–226.

Bruce, A. Y., F. Schulthess, and J. Mueke. 2009. Host Acceptance, Suitability, and Effects of Host Deprivation on the West African Egg Parasitoid Telenomus isis (Hymenoptera: Scelionidae) Reared on East African Stemborers Under Varying Temperature and Relative Humidity Regimens . Environ Entomol 38:904–919.

Bruning, K. 1991. Effects of temperature and light on the population dynamics of the Asterionella-Rhizophydium association. Journal of plankton research 13:707–719.

Bryla, D. R., T. J. Bouma, U. Hartmond, and D. M. Eissenstat. 2001. Influence of temperature and soil drying on respiration of individual roots in citrus: integrating greenhouse observations into a predictive model for the field. Plant, Cell and Environment 24:781–790.

Buitenhuis, E. T., T. Pangerc, D. J. Franklin, C. L. Quéré, and G. Malin. 2008. Growth rates of six coccolithophorid strains as a function of temperature. Limnol. Oceangr. 53:1181–1185.

Bunce, J. A. 2000. Acclimation of photosynthesis to temperature in eight cool and warm climate herbaceous C3 species: temperature dependence of parameters of a biochemical photosynthesis model. Photosynthesis Research 63:59–67.

Burnett, T. 1951. Effects of Temperature and Host Density on the Rate of Increase of an Insect Parasite. The American Naturalist 85:337–352.

Burton, A. J., and K. S. Pregitzer. 2003. Field measurements of root respiration indicate little to no seasonal temperature acclimation for sugar maple and red pine. Tree physiology 23:273–280.

Butterwick, C., S. Heaney, and J. Talling. 2005. Diversity in the influence of temperature on the growth rates of freshwater algae, and its ecological relevance. Freshwater Biology 50:291–300.

Caldwell, M. M., C. B. Osmond, and D. L. Nott. 1977. C4 Pathway Photosynthesis at Low Temperature in Cold-tolerant Atriplex Species. PLANT PHYSIOLOGY 60:157–164.

Cannon, J. 1993. Germination of the toxic dinoflagellate, Alexandrium minutum, from sediments in the Port River, South Australia. Toxic phytoplankton blooms in the sea. Elsevier. Amsterdam, The Netherlands pages 103–107.

Carey, R. W., and J. A. Berry. 1978. Effects of low temperature on respiration and uptake of rubidium ions by excised barley and corn roots. Plant physiology 61:858–860.

Casey, T. M. 1976. Activity Patterns, Body Temperature and Thermal Ecology in Two Desert Caterpillars (Lepidoptera: Sphingidae). Ecology 57:485–497.

Castro-Arevalo, M., M. Reyes-Diaz, M. Alberdi, V. Jara-Rodriguez, C. Sanhueza, L. J. Corcuera, and L. A. Bravo. 2008. Effects of low temperature acclimation on photosynthesis in three Chilean Proteaceae. Revista Chilena de Historia Natural 81:321–333.

Cen, Y.-P., and R. F. Sage. 2005. The regulation of Rubisco activity in response to variation in temperature and atmospheric CO2 partial pressure in sweet potato. Plant physiology 139:979–990.

Chabot, B. F., and J. F. Chabot. 1977. Effects of light and temperature on leaf anatomy and photosynthesis in Fragaria vesca. Oecologia 26:363–377.

Chadwick, L. E., and H. Rahn. 1954. Temperature Dependence of Rattling Frequency in the Rattlesnake, Crotalus v. viridis. Science 119:442–443.

Chang, F., R. G. Wear, and J. Reynolds. 1986. Effects of salinity, temperature, and light intensity on the growth rates of two halophilic phytoflagellates in mixed culture. New Zealand journal of marine and freshwater research 20:467–478.

Chapin III, F. S., and W. C. Oechel. 1983. Photosynthesis, respiration, and phosphate absorption by Carex aquatilis ecotypes along latitudinal and local environmental gradients. Ecology pages 743–751.

Chappell, P. D., and E. A. Webb. 2010. A molecular assessment of the iron stress response in the two phylogenetic clades of Trichodesmium . Environmental Microbiology 12:13–27.

Charles-Edwards, D., J. Charles-Edwards, and J. Cooper. 1971. The influence of temperature on photosynthesis and transpiration in ten temperate grass varieties grown in four different environments. Journal of Experimental Botany 22:650–662.

Chen, J., J. Shen, L. I. Hellgren, P. R. Jensen, and C. Solem. 2015. Adaptation of Lactococcus lactis to high growth temperature leads to a dramatic increase in acidification rate. Sci. Rep. 5:14199.

Chen, S., J. Wu, J. V. Huner, and R. F. Malone. 1995. Effects of temperature upon ablation-to-molt interval and mortality of red swamp crawfish (Procambarus clarkii) subjected to bilateral eyestalk ablation. Aquaculture 138:191–204.

Childs, M. R., and R. W. Clarkson. 1996. Temperature Effects on Swimming Performance of Larval and Juvenile Colorado Squawfish: Implications for Survival and Species Recovery. Transactions of the American Fisheries Society 125:940–947.

Chin, T., C. Chen, S. LIu, and S. Wu. 1965. INFLUENCE OF TEMPERATURE AND SALINITY ON THE GROWTH OF THREE SPECIES OF PLANKTONIC DIATOMS. Oceanologia Et Limnologia Sinica 4:002.

Chiverton, P. A. 1988. Searching behaviour and cereal aphid consumption by Bembidion lampros and Pterostichus cupreus, in relation to temperature and prey density. Entomologia Experimentalis et Applicata 47:173–182.

Chow, T., G. E. Long, and G. Tamaki. 1983. Effects of Temperature and Hunger on the Functional Response of Geocoris bullatus (Say) (Hemiptera: Lygaeidae) to Lygus spp. (Hemiptera: Miridae) Density. Environmental Entomology 12:1332–1338.

Christian, K. A., and C. R. Tracy. 1981. The effect of the thermal environment on the ability of hatchling Galapagos land iguanas to avoid predation during dispersal. Oecologia 49:218–223.

Claquin, P., I. Probert, S. Lefebvre, and B. Veron. 2008. Effects of temperature on photosynthetic parameters and TEP production in eight species of marine microalgae. Aquat. Microb. Ecol. 51:1–11.

Clopton, J. R. 2007. Temperature: Humans Regulating, Ants Conforming. The American Biology Teacher 69:e59–e63.

Coats, D., and M. Tyler. 1985. Encystment of the dinoflagellate Gyrodinium uncatenum: temperature and nutrient effects. j. Phycol 21:200–206.

Cockrell, B. J. 1984. Effects of Temperature and Oxygenation on Predator-Prey Overlap and Prey Choice of Notonecta glauca. The Journal of Animal Ecology 53:519.

COESEL, P. F., and K. WARDENAAR. 1990. Growth responses of planktonic desmid species in a temperaturelight gradient. Freshwater Biology 23:551–560.

Coles, S., and P. Jokiel. 1977. Effects of temperature on photosynthesis and respiration in hermatypic corals. Marine biology 43:209–216.

Collins, C. D., and C. W. Boylen. 1982. PHYSIOLOGICAL RESPONSES OF ANABAENA VARIABILIS (CYANOPHYCEAE) TO INSTANTANEOUS EXPOSURE TO VARIOUS COMBINATIONS OF LIGHT INTENSITY AND TEMPERATURE1. Journal of Phycology 18:206–211.

Conte, M. H., A. Thompson, D. Lesley, and R. P. Harris. 1998. Genetic and Physiological Influences on the Alkenone/Alkenoate Versus Growth Temperature Relationship in Emiliania huxleyi and Gephyrocapsa Oceanica. Geochimica et Cosmochimica Acta 62:51–68.

Cook, J. 1966. Adaptations to temperature in two closely related strains of Euglena gracilis. Biological Bulletin 131:83–93.

Cooper, W. 2000. EFFECT OF TEMPERATURE ON ESCAPE BEHAVIOUR BY AN ECTOTHERMIC VERTEBRATE, THE KEELED EARLESS LIZARD (HOLBROOKIA PROPINQUA). Behaviour 137:1299–1315.

Covey-Crump, E. M., R. G. Attwood, and O. K. Atkin. 2002. Regulation of root respiration in two species of Plantago that differ in relative growth rate: the effect of short- and long-term changes in temperature. Plant, Cell and Environment 25:1501–1513.

Cowling, S., and R. Sage. 1998. Interactive effects of low atmospheric CO2 and elevated temperature on growth, photosynthesis and respiration in Phaseolus vulgaris. Plant, Cell & Environment 21:427–435.

Criddle, R. S., B. N. Smith, and L. D. Hansen. 1997. A respiration based description of plant growth rate responses to temperature. Planta 201:441–445.

Crist, T. O., and J. A. MacMahon. 1991. Foraging Patterns of Pogonomyrmex occidentalis (Hymenoptera: Formicidae) in a Shrub-Steppe Ecosystem: The Roles of Temperature, Trunk Trails, and Seed Resources. Environmental Entomology 20:265–275.

Crocker, R. L., W. H. Whitcomb, and R. M. Ray. 1975. Effects of Sex, Developmental Stage, and Temperature on Predation by Geocoris punctipes. Environmental Entomology 4:531–534.

Csavina, J., B. Stuart, R. Guy Riefler, and M. Vis. 2011. Growth optimization of algae for biodiesel production. Journal of applied microbiology 111:312–318.

Curl, H., and G. C. McLeod. 1961. THE PHYSIOLOGICAL ECOLOGY OF A MARINE DIATOM, SKELETONEMA-COSTATUM (GREV) CLEVE. Journal of Marine Research 19:70–88.

Damme, R. V., D. Bauwens, A. M. Castilla, and R. F. Verheyen. 1989. Altitudinal variation of the thermal biology and running performance in the lizard Podarcis tiliguerta. Oecologia 80:516–524.

Dauta, A. 1982. Conditions de développement du phytoplancton. Etude comparative du comportement de huit espèces en culture. I. Détermination des paramètres de croissance en fonction de la lumière et de la température. Pages 217–262 *in* Annales de limnologie. Vol. 18. Cambridge Univ Press.

Dauta, A., J. Devaux, F. Piquemal, and L. Boumnich. 1990. Growth rate of four freshwater algae in relation to light and temperature. Hydrobiologia 207:221–226.

Davey, K. 1994. Modelling the combined effect of temperature and pH on the rate coefficient for bacterial growth. International Journal of Food Microbiology 23:295–303.

Davison, I. R. 1987. ADAPTATION OF PHOTOSYNTHESIS IN LAMINARIA SACCHARINA (PHAEOPHYTA) TO CHANGES IN GROWTH TEMPERATURE. Journal of Phycology 23:273–283.

Davison, I. R., R. Greene, and E. Podolak. 1991. Temperature acclimation of respiration and photosynthesis in the brown algaLaminaria saccharina. Marine Biology 110:449–454.

Dawes, C. J., R. E. Moon, and M. A. Davis. 1978. The photosynthetic and respiratory rates and tolerances of benthic algae from a mangrove and salt marsh estuary: A comparative study. Estuarine and Coastal Marine Science 6:175–185.

de Boer, M. K. 2004. Temperature responses of three Fibrocapsa japonica strains (Raphidophyceae) from different climate regions. Journal of Plankton Research 27:47–60.

de Vries, F. P., J. Witlage, and D. Kremer. 1979. Rates of respiration and of increase in structural dry matter in young wheat, ryegrass and maize plants in relation to temperature, to water stress and to their sugar content. Annals of Botany 44:595–609.

Delucia, E. H. 1986. Effect of low root temperature on net photosynthesis, stomatal conductance and carbohydrate concentration in Engelmann spruce (Picea engelmannii Parry ex Engelm.) seedlings. Tree Physiology 2:143–154.

Denius, H. R., and P. H. Homann. 1972. The relation between photosynthesis, respiration, and Crassulacean acid metabolism in leaf slices of Aloe arborescens Mill. Plant physiology 49:873–880.

Derlinden, E. V., and J. V. Impe. 2012. Modeling growth rates as a function of temperature: Model performance evaluation with focus on the suboptimal temperature range. International Journal of Food Microbiology 158:73–78.

Dermoun, D., D. Chaumont, J.-M. Thebault, and A. Dauta. 1992. Modelling of growth of Porphyridium cruentum in connection with two interdependent factors: light and temperature. Bioresource technology 42:113–117.

Dmi'el, R., and D. Rappeport. 1976. Effect of temperature on metabolism during running in the lizard Uromastix aegyptius. Physiological Zoology pages 77–84.

Doemel, W. N., and T. D. Brock. 1970. The upper temperature limit of Cyanidium caldarium. Archiv für Mikrobiologie 72:326–332.

Donk, E., and S. S. Kilham. 1990. TEMPERATURE EFFECTS ON SILICON-AND PHOSPHORUS-LIMITED GROWTH AND COMPETITIVE INTERACTIONS AMONG THREE DIATOMS. Journal of Phycology 26:40–50.

Downton, W. J. S., J. A. Berry, and J. R. Seemann. 1984. Tolerance of photosynthesis to high temperature in desert plants. Plant Physiology 74:786–790.

Dreisig, H. 1981. The Rate of Predation and Its Temperature Dependence in a Tiger Beetle, Cicindela Hybrida. Oikos 36:196.

Drent, W. J., G. A. Lahpor, W. M. Wiegant, and J. C. Gottschal. 1991. Fermentation of inulin by Clostridium thermosuccinogenes sp. nov., a thermophilic anaerobic bacterium isolated from various habitats. Applied and environmental microbiology 57:455–462.

Drew, E. A. 1977. The physiology of photosynthesis and respiration in some Antarctic marine algae. Br Antarct Surv Bull 46:59–76.

———. 1979. Physiological aspects of primary production in seagrasses. Aquatic Botany 7:139–150.

Dreyer, E., X. Le Roux, P. Montpied, F. A. Daudet, and F. Masson. 2001. Temperature response of leaf photosynthetic capacity in seedlings from seven temperate tree species. Tree Physiology 21:223–232.

Dungan, R. J., D. Whitehead, and R. P. Duncan. 2003. Seasonal and temperature dependence of photosynthesis and respiration for two co-occurring broad-leaved tree species with contrasting leaf phenology. Tree Physiology 23:561–568.

Durbin, E. G. 1974. STUDIES ON THE AUTECOLOGY OF THE MARINE DIATOM THALASSIOSIRA NORDENSKIOLDII CLEVE. 1. THE INFLUENCE OF DAYLENGTH, LIGHT INTENSITY, AND TEMPERATURE ON GROWTH1. Journal of Phycology 10:220–225.

Dyer, D. L., and R. D. Gafford. 1961. Some characteristics of a thermophilic blue-green alga. Science 134:616–617.

Eddy, B. 1956. The suitability of some algae for mass cultivation for food, with special reference to Dunaliella bioculata. Journal of Experimental Botany 7:372–380.

Edvardsen, B., and E. Paasche. 1992. TWO MOTILE STAGES OF CHRYSOCHROMULINA POLYLEPIS (PRYMNESIOPHYCEAE): MORPHOLOGY, GROWTH, AND TOXICITY. Journal of phycology 28:104–114.

Edwards, D. J. 1971. Effect of temperature on rate of passage of food through the alimentary canal of the plaice Pleuronectes platessa L. Journal of Fish Biology 3:433–439.

Eggert, A., and C. Wiencke. 2000. Adaptation and acclimation of growth and photosynthesis of five Antarctic red algae to low temperatures. Polar Biology 23:609–618.

Eggleston, D. B. 1990. Behavioural Mechanisms Underlying Variable Functional Responses of Blue Crabs, Callinectes sapidus Feeding on Juvenile Oysters, Crassostrea virginica. The Journal of Animal Ecology 59:615.

Ehleringer, J. R., and O. Björkman. 1978. A comparison of photosynthetic characteristics of Encelia species possessing glabrous and pubescent leaves. Plant physiology 62:185–190.

El-Sabaawi, R., and P. J. Harrison. 2006. INTERACTIVE EFFECTS OF IRRADIANCE AND TEMPERATURE ON THE PHOTOSYNTHETIC PHYSIOLOGY OF THE PENNATE DIATOM PSEUDO-NITZSCHIA GRANII (BACILLARIOPHYCEAE) FROM THE NORTHEAST SUBARCTIC PACIFIC . Journal of Phycology 42:778–785.

ELLIOTT, J. M. 1972. Rates of gastric evacuation in brown trout, Salmo trutta L. Freshwater Biology 2:1–18.

Elner, R., and R. N. Hughes. 1978. Energy maximization in the diet of the shore crab, Carcinus maenas. The Journal of Animal Ecology pages 103–116.

Elner, R. W. 1980. The influence of temperature, sex and chela size in the foraging strategy of the shore crab, Carcinus maenas (L.) . Marine Behaviour and Physiology 7:15–24.

Else, P., and A. Bennett. 1987. The thermal dependence of locomotor performance and muscle contractile function in the salamander Ambystoma tigrinum nebulosum. Journal of Experimental Biology 128:219–233.

Emerson, R., and L. Green. 1934. Manometric measurements of photosynthesis in the marine alga Gigartina. The Journal of general physiology 17:817.

Esener, A. A., J. A. Roels, and N. W. F. Kossen. 1981. The influence of temperature on the maximum specific growth rate ofKlebsiella pneumoniae. Biotechnol. Bioeng. 23:1401–1405.

Evans, A. S., K. L. Webb, and P. A. Penhale. 1986. Photosynthetic temperature acclimation in two coexisting seagrasses, Zostera marina L. and Ruppia maritima L. Aquatic Botany 24:185–197.

Falcón, L., S. Pluvinage, and E. Carpenter. 2005. Growth kinetics of marine unicellular N2-fixing cyanobacterial isolates in continuous culture in relation to phosphorus and temperature. Marine Ecology Progress Series 285:3–9.

Falkowski, P. G. 1977. The adenylate energy charge in marine phytoplantkon: The effect of temperature on the physiological state of Skeletonema costatum (Grev.) Cleve. Journal of Experimental Marine Biology and Ecology 27:37–45.

Fan, L., A. Vonshak, and S. Boussiba. 1994. EFFECT OF TEMPERATURE AND IRRADIANCE ON GROWTH OF HAEMATOCOCCUS PLUVIALIS (CHLOROPHYCEAE). Journal of Phycology 30:829–833.

Fang, T., Y. Liu, and L. Huang. 2013. Growth kinetics of Listeria monocytogenes and spoilage microorganisms in fresh-cut cantaloupe. Food Microbiology 34:174–181.

Fawley, M. W. 1984. EFFECTS OF LIGHT INTENSITY AND TEMPERATURE INTERACTIONS ON GROWTH CHARACTERISTICS OF PHAEODACTYLUM TRICORNUTUM (BACILLARIOPHYCEAE)1. Journal of Phycology 20:67–72.

FEDORENKO, A. Y. 1975. Feeding characteristics and predation impact of Chaoborus (Diptera, Chaoboridae) larvae in a small lake. Limnol. Oceangr. 20:250–258.

Feil, H., and A. H. Purcell. 2001. Temperature-Dependent Growth and Survival of Xylella fastidiosa in Vitro and in Potted Grapevines . Plant Disease 85:1230–1234.

Feng, S., and W. V. Winkle. 1975. The effect of temperature and salinity on the heart beat of Crassostrea virginica. Comparative Biochemistry and Physiology Part A: Physiology 50:473–476.

Ferguson, T. J., and R. A. Mah. 1983. Isolation and characterization of an H2-oxidizing thermophilic methanogen. Applied and environmental microbiology 45:265–274.

Fiala, M., and L. Oriol. 1990. Light-temperature interactions on the growth of Antarctic diatoms. Polar Biol 10:629–636.

Flinn, P., and D. Hagstrum. 2002. Temperature-mediated functional response of Theocolax elegans (Hymenoptera: Pteromalidae) parasitizing Rhyzopertha dominica (Coleoptera: Bostrichidae) in stored wheat. Journal of Stored Products Research 38:185–190.

Flinn, P. W. 1991. Temperature-Dependent Functional Response of the Parasitoid Cephalonomia waterstoni (Gahan) (Hymenoptera: Bethylidae) Attacking Rusty Grain Beetle Larvae (Coleoptera: Cucujidae). Environmental Entomology 20:872–876.

Foerster, L. A., M. do R.F. Avanci, and A. K. Doetzer. 1999. Effect of temperature on the development and progeny production of Glyptapanteles muesebecki (Blanchard) (Hymenoptera: Braconidae) parasitizing larvae of Pseudaletia sequax Franclemont (Lepidoptera: Noctuidae). An. Soc. Entomol. Bras. 28:243–249.

Fogg, G. E., and B. Thake. 1987. Algal cultures and phytoplankton ecology. Univ of Wisconsin Press.

Foy, R. 1983. Interaction of temperature and light on the growth rates of two planktonic Oscillatoria species under a short photoperiod regime. British phycological journal 18:267–273.

Foy, R., and C. Gibson. 1993. The influence of irradiance, photoperiod and temperature on the growth kinetics of three planktonic diatoms. European Journal of Phycology 28:203–212.

Foy, R., C. Gibson, and R. Smith. 1976. The influence of daylength, light intensity and temperature on the growth rates of planktonic blue-green algae. British phycological journal 11:151–163.

Foyle, T. P., R. K. O'dor, and R. W. ELNER. 1989. Energetically defining the thermal limits of the snow crab. Journal of Experimental Biology 145:371–393.

FRANTZ, J. M. 2004. Night Temperature has a Minimal Effect on Respiration and Growth in Rapidly Growing Plants. Annals of Botany 94:155–166.

Franzmann, P., C. Haddad, R. Hawkes, W. Robertson, and J. Plumb. 2005. Effects of temperature on the rates of iron and sulfur oxidation by selected bioleaching Bacteria and Archaea: Application of the Ratkowsky equation. Minerals Engineering 18:1304–1314.

Fuiman, L., and D. Ottey. 1993. Temperature effects on spontaneous behavior of larval and juvenile red drum Sciaenops ocellatus, and implications for foraging. Fishery Bulletin 91:23–35.

Fuiman, L. A. 1986. Burst-Swimming Performance of Larval Zebra Danios and the Effects of Diel Temperature Fluctuations. Transactions of the American Fisheries Society 115:143–148.

———. 1991. Influence of temperature on evasive responses of Atlantic herring larvae attacked by yearling herring, Clupea harengus L. Journal of Fish Biology 39:93–102.

Fuiman, L. A., and R. S. Batty. 1994. Susceptibility of Atlantic herring and plaice larvae to predation by juvenile cod and herring at two constant temperatures. Journal of Fish Biology 44:23–34.

Gallepp, G. W. 1977. Responses of Caddisfly Larvae (Brachycentrus spp.) to Temperature, Food Availability and Current Velocity. American Midland Naturalist 98:59.

Gilchrist, G. W. 1996. A Quantitative Genetic Analysis of Thermal Sensitivity in the Locomotor Performance Curve of Aphidius ervi. Evolution 50:1560.

Goldasteh, S., A. Talebi, Y. Fathipour, H. Ostovan, A. Zamani, and V. Shoushtari. 2009. Effect of temperature on life history and population growth parameters of Planococcus citri (Homoptera, Pseudococcidae) on coleus [Solenostemon scutellarioides (L.) Codd.]. Arch. biol. sci. (Beogr.) 61:329–336.

Goldman, J. C., and E. J. Carpenter. 1974. A kinetic approach to the effect of temperature on algal growth. Limnology and Oceanography 19:756–766.

González, J. M., C. Kato, and K. Horikoshi. 1995. Thermococcus peptonophilus sp. nov., a fast-growing, extremely thermophilic archaebacterium isolated from deep-sea hydrothermal vents. Archives of Microbiology 164:159–164.

González, J. M., Y. Masuchi, F. T. Robb, J. W. Ammerman, D. L. Maeder, M. Yanagibayashi, J. Tamaoka, and C. Kato. 1998. Pyrococcus horikoshii sp. nov., a hyperthermophilic archaeon isolated from a hydrothermal vent at the Okinawa Trough. Extremophiles 2:123–130.

Grafius, E., and F. W. Warner. 1989. Predation by Bembidion quadrimaculatum (Coleoptera: Carabidae) on Delia antiqua (Diptera: Anthomyiidae). Environmental Entomology 18:1056–1059.

GRAHAM, J. M., P. ARANCIBIA-AVILA, and L. E. GRAHAM. 1996. Physiological ecology of a species of the filamentous green alga Mougeotia under acidic conditions: Light and temperature effects on photosynthesis and respiration. Limnol. Oceangr. 41:253–262.

Graham, J. M., M. T. Auer, R. P. Canale, and J. P. Hoffmann. 1982. Ecological Studies and Mathematical Modeling of Cladophora in Lake Huron: 4. Photosynthesis and Respiration as Functions of Light and Temperature. Journal of Great Lakes Research 8:100–111.

Graham, W. D., J. E. Thorpe, and N. B. Metcalfe. 1996. Seasonal current holding performance of juvenile Atlantic salmon in relation to temperature and smolting. Can. J. Fish. Aquat. Sci. 53:80–86.

Gratani, L., P. Pesoli, M. Crescente, K. Aichner, and W. Larcher. 2000. Photosynthesis as a temperature indicator in Quercus ilex L. Global and Planetary Change 24:153–163.

Gratani, L., L. Varone, and R. Catoni. 2008. Relationship between net photosynthesis and leaf respiration in Mediterranean evergreen species. Photosynthetica 46:567–573.

Gray, J. 1923. The Mechanism of Ciliary Movement. III.–The Effect of Temperature. Proceedings of the Royal Society B: Biological Sciences 95:6–15.

Gresens, S. E., M. L. Cothran, and J. H. Thorp. 1982. The influence of temperature on the functional response of the dragonfly Celithemis fasciata (Odonata: Libellulidae). Oecologia 53:281–284.

Grimm, N., and T. Weisse. 1985. Die Temperaturabhängigkeit des Wachstums vonPhaeocystis pouchetii (Haptophyceae) in Batchkulturen. Helgoländer Meeresuntersuchungen 39:201–211.

Grote, R., L. Li, J. Tamaoka, C. Kato, K. Horikoshi, and G. Antranikian. 1999. Thermococcus siculi sp. nov., a novel hyperthermophilic archaeon isolated from a deep-sea hydrothermal vent at the Mid-Okinawa Trough. Extremophiles 3:55–62.

Grzebyk, D., and B. Berland. 1996. Influences of temperature, salinity and irradiance on growth of Prorocentrum minimum (Dinophyceae) from the Mediterranean Sea . J Plankton Res 18:1837–1849.

Guillard, R. R. L., and J. H. Ryther. 1962. STUDIES OF MARINE PLANKTONIC DIATOMS: I. CYCLOTELLA NANA HUSTEDT, AND DETONULA CONFERVACEA (CLEVE) GRAN. Can. J. Microbiol. 8:229–239.

Hailety, A., and P. M. C. Davies. 2009. Effects of size, sex, temperature and condition on activity metabolism and defence behaviour of the viperine snake, Natrix maura. Journal of Zoology 208:541–558.

HALDIMANN, P., and U. FELLER. 2004. Inhibition of photosynthesis by high temperature in oak (Quercus pubescens L.) leaves grown under natural conditions closely correlates with a reversible heat-dependent reduction of the activation state of ribulose-1,5-bisphosphate carboxylase/oxygenase. Plant, Cell and Environment 27:1169–1183.

Hällgren, J.-E., E. Sundbom, and M. Strand. 1982. Photosynthetic responses to low temperature in Betula pubescens and Betula tortuosa. Physiologia plantarum 54:275–282.

Hamilton-Brehm, S. D., J. J. Mosher, T. Vishnivetskaya, M. Podar, S. Carroll, S. Allman, T. J. Phelps, M. Keller, and J. G. Elkins. 2009. Caldicellulosiruptor obsidiansis sp. nov., an Anaerobic, Extremely Thermophilic, Cellulolytic Bacterium Isolated from Obsidian Pool, Yellowstone National Park. Applied and Environmental Microbiology 76:1014–1020.

Hancke, K., T. B. Hancke, L. M. Olsen, G. Johnsen, and R. N. Glud. 2008. TEMPERATURE EFFECTS ON MICROALGAL PHOTOSYNTHESIS-LIGHT RESPONSES MEASURED BY O2 PRODUCTION, PULSE-AMPLITUDE-MODULATED FLUORESCENCE, AND 14C ASSIMILATION1. Journal of Phycology 44:501–514.

Harley, P., J. Tenhunen, and O. Lange. 1986. Use of an analytical model to study limitations on net photosynthesis in Arbutus unedo under field conditions. Oecologia 70:393–401.

Harley, P., J. Weber, and D. M. Gates. 1985. Interactive effects of light, leaf temperature, CO2 and O2 on photosynthesis in soybean. Planta 165:249–263.

Harley, P. C., J. D. Tenhunen, K. J. Murray, and J. Beyers. 1989. Irradiance and temperature effects on photosynthesis of tussock tundra Sphagnum mosses from the foothills of the Philip Smith Mountains, Alaska. Oecologia 79:251–259.

HARLEY, P. C., R. B. THOMAS, J. F. REYNOLDS, and B. R. STRAIN. 1992. Modelling photosynthesis of cotton grown in elevated CO2. Plant, Cell and Environment 15:271–282.

Healey, F. 1983. Effect of temperature and light intensity on the growth rate of Synura sphagnicola. Journal of plankton research 5:767–774.

Heckrotte, C. 1967. Relations of Body Temperature, Size, and Crawling Speed of the Common Garter Snake, Thamnophis s. sirtalis. Copeia 1967:759.

Heiman, D. R., and A. W. Knight. 1975. The influence of temperature on the bioenergetics of the carnivorous stonefly nymph, Acroneuria californica Banks (Plecoptera: Perlidae). Ecology pages 105–116.

Hellmuth, E. 1967. A method of determining true values for photosynthesis and respiration under field conditions. Flora B 157:265–286.

Hellmuth, E. O. 1971. Eco-Physiological Studies on Plants in Arid and Semi-Arid Regions in Western Australia: III. Comparative Studies on Photosynthesis, Respiration and Water Relations of Ten Arid Zone and Two Semi-Arid Zone Plants Under Winter and Late Summer Climatic Conditions. The Journal of Ecology pages 225–259.

Hertz, P. E., R. B. Huey, and E. Nevo. 1982. Fight versus flight: Body temperature influences defensive responses of lizards. Animal Behaviour 30:676–679.

Hikosaka, K., A. Murakami, and T. Hirose. 1999. Balancing carboxylation and regeneration of ribulose-1,5-bisphosphate in leaf photosynthesis: temperature acclimation of an evergreen tree, Quercus myrsinaefolia. Plant, Cell and Environment 22:841–849.

HIRANO, M., and L. C. ROME. 1984. Jumping performance of frogs (Rana pipiens) as a function of muscle temperature. Journal of Experimental Biology 108:429–439.

Hocutt, C. H. 1973. Swimming Performance of Three Warmwater Fishes Exposed to a Rapid Temperature Change. Chesapeake Science 14:11.

Hodaifa, G., M. E. Martínez, and S. Sánchez. 2010. Influence of temperature on growth of Scenedesmus obliquus in diluted olive mill wastewater as culture medium. Engineering in Life Sciences 10:257–264.

Holaday, A. S., W. Martindale, R. Alred, A. L. Brooks, and R. C. Leegood. 1992. Changes in Activities of Enzymes of Carbon Metabolism in Leaves during Exposure of Plants to Low Temperature. PLANT PHYSIOLOGY 98:1105–1114.

Holt, S. J. 1955. On the Foraging Activity of the Wood Ant. The Journal of Animal Ecology 24:1.

Huang, L., A. Hwang, and J. Phillips. 2011. Effect of Temperature on Microbial Growth Rate-Mathematical Analysis: The Arrhenius and Eyring-Polanyi Connections. Journal of Food Science 76:E553–E560.

Huang, X. 2005. Interactive effects of soil temperature and moisture on Concord grape root respiration. Journal of Experimental Botany 56:2651–2660.

Huang, X., C. Li, C. Liu, and D. Zeng. 2001. Studies on the Ecological Factors of Oocystis borgei. Journal of Zhanjiang Ocean University 22:8–12.

Huey, R. B., P. H. Niewiarowski, J. Kaufmann, and J. C. Herron. 1989. Thermal Biology of Nocturnal Ectotherms: Is Sprint Performance of Geckos Maximal at Low Body Temperatures? Physiological Zoology 62:488–504.

HUEY, R. B., and R. STEVENSON. 1979. Integrating Thermal Physiology and Ecology of Ectotherms: A Discussion of Approaches. American Zoologist 19:357–366.

Hulburt, E. 1982. The adaptation of marine phytoplankton species to nutrient and temperature. Ocean science and engineering (USA) .

Hulburt, E. M., and R. R. Guillard. 1968. The relationship of the distribution of the diatom Skeletonema tropicum to temperature. Ecology pages 337–339.

Hull, L. A., D. Asquith, and P. D. Mowery. 1977. The Functional Responses of Stethorus punctum to Densities of the European Red Mite. Environmental Entomology 6:85–90.

HURLBERT, A. H., F. BALLANTYNE, and S. POWELL. 2008. Shaking a leg and hot to trot: the effects of body size and temperature on running speed in ants. Ecological Entomology 33:144–154.

Huser, B. A., K. Wuhrmann, and A. J. B. Zehnder. 1982. Methanothrix soehngenii gen. nov. sp. nov., a new acetotrophic non-hydrogen-oxidizing methane bacterium. Archives of Microbiology 132:1–9.

Ignatiades, L., and T. J. Smayda. 1970. AUTECOLOGICAL STUDIES ON THE MARINE DIATOM RHIZOSOLENIA FRAGILISSIMA BERGON. I. THE INFLUENCE OF LIGHT, TEMPERATURE, AND SALINITY. Journal of Phycology 6:332–339.

Imamura, T., J. Uraichuen, P. Visarathanonth, S. Morimoto, and A. Miyanoshita. 2004. Effect of temperature on development of Theocolax elegans (Westwood) (Hymenoptera: Pteromalidae) parasitizing larvae of the maize weevil Sitophilus zeamais (Coleoptera: Curculionidae) in brown rice. Applied Entomology and Zoology 39:497–503.

Inoue, N., Y. Taira, T. Emi, Y. Yamane, Y. Kashino, H. Koike, and K. Satoh. 2001. Acclimation to the growth temperature and the high-temperature effects on photosystem II and plasma membranes in a mesophilic cyanobacterium, Synechocystis sp. PCC6803. Plant and Cell Physiology 42:1140–1148.

Isaksen, M., and B. Jorgensen. 1996. Adaptation of psychrophilic and psychrotrophic sulfate-reducing bacteria to permanently cold marine environments. Applied and Environmental Microbiology 62:408–414.

James, C., S. Al-Hinty, and A. Salman. 1989. Growth and $\omega 3$ fatty acid and amino acid composition of microalgae under different temperature regimes. Aquaculture 77:337–351.

Jensen, M. Ø., and Ø. Moestrup. 1997. Autecology of the toxic dinoflagellate Alexandrium ostenfeldii : life history and growth at different temperatures and salinities . European Journal of Phycology 32:9–18.

Jobling, M., and P. S. Davies. 2006. Gastric evacuation in plaice, Pleuronectes platessa L.: effects of temperature and meal size. Journal of Fish Biology 14:539–546.

Johnson, T., and A. Bennett. 1995. The thermal acclimation of burst escape performance in fish: an integrated study of molecular and cellular physiology and organismal performance. The Journal of experimental biology 198:2165–2175.

Johnson, Z. I. 2006. Niche Partitioning Among Prochlorococcus Ecotypes Along Ocean-Scale Environmental Gradients. Science 311:1737–1740.

Johnston, A. 1996. The effect of environmental variables on 13C discrimination by two marine phytoplankton. Marine Ecology Progress Series 132:257–263.

Johnston, T. A., and J. A. Mathias. 1994. The effects of temperature on feeding in zooplanktivorous walleye, Stizostedion vitreum, larvae. Environmental biology of fishes 40:189–198.

Jolliffe, P., and E. Tregunna. 1968. Effect of temperature, CO2 concentration, and light intensity on oxygen inhibition of photosynthesis in wheat leaves. Plant physiology 43:902–906.

Jørgensen, C. B. 1974. On gill function in the mussel Mytilus Edulis L. . Ophelia 13:187–232.

Jørgensen, C. B., P. S. Larsen, and H. U. Riisgård. 1990. Effects of temperature on the mussel pump. Marine Ecology-Progress Series 64:89–97.

Jr., W. W., A. Bianchini, C. C. Sanchez, and L. H. Poersch. 2003. The effect of temperature, salinity and nitrogen products on food consumption of pink shrimp Farfantepenaeus paulensis. Brazilian Archives of Biology and Technology 46:135–141.

Juhl, A. R. 2005. Growth rates and elemental composition of Alexandrium monilatum, a red-tide dinoflagellate. Harmful Algae 4:287–295.

Kaeriyama, H., E. Katsuki, M. Otsubo, M. Yamada, K. Ichimi, K. Tada, and P. J. Harrison. 2011. Effects of temperature and irradiance on growth of strains belonging to seven Skeletonema species isolated from Dokai Bay, southern Japan . European Journal of Phycology 46:113–124.

Kahn, S., O. A. &, and Y. Onoue. 1998. Physiological investigations of a neurotoxin-producing phytoflagellate, Chattonella marina (Raphidophyceae). Aquaculture Research 29:9–17.

Kalyebi, A., W. Overholt, F. Schulthess, J. Mueke, and S. Sithanantham. 2006. The effect of temperature and humidity on the bionomics of six African egg parasitoids (Hymenoptera: Trichogrammatidae). Bull. Entomol. Res. 96:305–314.

Karentz, D., and T. Smayda. 1984. Temperature and seasonal occurrence patterns of 30 dominant phytoplankton species in Narragansett Bay over a 22-year period (1959-1980). Marine Ecology Progress Series 18:277–293.

Kasai, F., and T. Ichimura. 1990. Temperature optima of three closely related mating groups of the Closterium ehrenbergii (Chlorophyta) species complex. Phycologia 29:396–402.

Khan, S., O. Arakawa, and Y. Onoue. 1996. Growth Characteristics of a Neurotoxin-Producing Chloromonad Fibrocapsa japonica (Raphidophyceae). J World Aquaculture Soc 27:247–253.

Kim, J. H., M. O. Shin, K. L. Lee, and H. S. Kim. 2008. Effect of environmental conditions on the growth of Synura petersenii (Synurophyceae) in vitro and two eutrophic water bodies in Korea. Nova Hedwigia 86:529–544.

Kim, K. 2005. Temperature Dependence of Photosynthesis in Arabidopsis Plants with Modifications in Rubisco Activase and Membrane Fluidity. Plant and Cell Physiology 46:522–530.

KIM, S., D. GITZ, R. SICHER, J. BAKER, D. TIMLIN, and V. REDDY. 2007. Temperature dependence of growth, development, and photosynthesis in maize under elevated CO2. Environmental and Experimental Botany 61:224–236.

KISHI, D., M. MURAKAMI, S. NAKANO, and K. MAEKAWA. 2005. Water temperature determines strength of top-down control in a stream food web. Freshwater Biology 50:1315–1322.

Kishimoto, N., Y. Ohnishi, M. Ohnishi, and I. Somiya. 1998. Effects of water temperature and light intensity on growth rate and death rate of freshwater dinoflagellate Peridinium bipes. Japanese Journal of Limnology (Japan) .

Kitaya, Y., L. Xiao, A. Masuda, T. Ozawa, M. Tsuda, and K. Omasa. 2008. Effects of temperature, photosynthetic photon flux density, photoperiod and O2 and CO2 concentrations on growth rates of the symbiotic dinoflagellate, Amphidinium sp. Journal of Applied Phycology 20:737–742.

Kittner, C., and H. Riisgård. 2005. Effect of temperature on filtration rate in the mussel Mytilus edulis: no evidence for temperature compensation. Marine Ecology Progress Series 305:147–152.

Kivivuori, L. 1983. Temperature acclimation of walking in the crayfish Astacus astacus L. Comparative Biochemistry and Physiology Part A: Physiology 75:375–378.

Knoblauch, C., and B. B. Jorgensen. 1999. Effect of temperature on sulphate reduction, growth rate and growth yield in five psychrophilic sulphate-reducing bacteria from Arctic sediments. Environ Microbiol 1:457–467.

Knowles, T. W., and P. D. Weigl. 1990. Thermal Dependence of Anuran Burst Locomotor Performance. Copeia 1990:796.

Komarek, J., and J. Ruzicka. 1969. Effect of temperature on the growth and variability of Scenedesmus quadricauda (Turp.) BrEb. Studies in Phycology. Academia, Prague pages 262–292.

Konai, M., E. A. Clark, M. Camp, A. L. Koeh, and R. F. Whitcomb. 1996. Temperature Ranges, Growth Optima, and Growth Rates of Spiroplasma (Spiroplasmataceae , class Mollicutes) Species. Current Microbiology 32:314–319.

Konopka, A., and T. D. Brock. 1978. Effect of temperature on blue-green algae (cyanobacteria) in Lake Mendota. Applied and Environmental Microbiology 36:572–576.

Kotsyurbenko, O. R., M. V. Simankova, A. N. Nozhevnikova, T. N. Zhilina, N. P. Bolotina, A. M. Lysenko, and G. A. Osipov. 1995. New species of psychrophilic acetogens: Acetobacterium bakii sp. nov., A. paludosum sp. nov., A. fimetarium sp. nov. Archives of Microbiology 163:29–34.

Kratz, W. A., and J. Myers. 1955. Nutrition and growth of several blue-green algae. American Journal of Botany pages 282–287.

Krawiec, R. W. 1982. Autecology and clonal variability of the marine centric diatom Thalassiosira rotula (Bacillariophyceae) in response to light, temperature and salinity. Marine Biology 69:79–89.

Kriedemann, P. 1968. Photosynthesis in vine leaves as a function oflight intensity, temperature, and leaf age. Vitis 7:213–220.

Krist, K. 1997. Description and mechanisms of bacterial growth responses to water activity and compatible solutes. Ph.D. thesis. University of Tasmania.

———. 1998. Final optical density and growth rate; effects of temperature and NaCl differ from acidity. International Journal of Food Microbiology 43:195–203.

Krüger, G., and J. Eloff. 1978. The effect of temperature on specific growth rate and activation energy of Microcystis and Synechococcus isolates relevant to the onset of natural blooms. Journal of the Limnological Society of southern Africa 4:9–20.

Krugner, R., K. M. Daane, A. B. Lawson, and G. Y. Yokota. 2007. Temperature-dependent development of Macrocentrus iridescens (Hymenoptera: Braconidae) as a parasitoid of the obliquebanded leafroller (Lepidoptera: Tortricidae): Implications for field synchrony of parasitoid and host. Biological Control 42:110–118.

Ku, S.-B., G. E. Edwards, and C. B. Tanner. 1977. Effects of light, carbon dioxide, and temperature on photosynthesis, oxygen inhibition of photosynthesis, and transpiration in Solanum tuberosum. Plant Physiology 59:868–872.

Kubien, D. S. 2003. C4 Photosynthesis at Low Temperature. A Study Using Transgenic Plants with Reduced Amounts of Rubisco. PLANT PHYSIOLOGY 132:1577–1585.

Kübler, J. E., and I. R. Davison. 1993. High-temperature tolerance of photosynthesis in the red alga Chondrus crispus. Marine Biology 117:327–335.

Kuebler, J. E., I. R. Davison, and C. Yarish. 1991. Photosynthetic adaptation to temperature in the red algae Lomentaria baileyana and Lomentaria orcadensis . British Phycological Journal 26:9–19.

Kumar, D., and L. L. Tieszen. 1980. Photosynthesis in Coffea arabica. I. Effects of light and temperature. Experimental Agriculture 16:13–19.

Kuo, M.-H., W.-N. Lu, M. C. Chiu, Y.-H. Kuo, and S.-H. Hwang. 2006. Temperature-dependent development and population growth of Tetraneura nigriabdominalis (Homoptera: Pemphigidae) on three host plants. Journal of economic entomology 99:1209–1213.

Labate, C. A., and R. C. Leegood. 1988. Limitation of photosynthesis by changes in temperature. Planta 173:519–527.

Langdon, C. 1987. On the causes of interspecific differences in the growth-irradiance relationship for phytoplankton. Part I. A comparative study of the growth-irradiance relationship of three marine phytoplankton species: Skeletonema costatum, Olisthodiscus luteus and Gonyaulax tamarensis. Journal of Plankton Research 9:459–482.

Lange, O. L., J. Belnap, and H. Reichenberger. 1998. Photosynthesis of the cyanobacterial soil-crust lichen Collema tenax from arid lands in southern Utah, USA: role of water content on light and temperature responses of CO2 exchange. Functional Ecology 12:195–202.

Lange, O. L., and T. G. A. Green. 2004. Lichens show that fungi can acclimate their respiration to seasonal changes in temperature. Oecologia 142:11–19.

Larimore, R. W., and M. J. Duever. 1968. Effects of Temperature Acclimation on the Swimming Ability of Smallmouth Bass Fry. Transactions of the American Fisheries Society 97:175–184.

Larsen, A., S. Bryant, and U. Båmstedt. 1998. Growth rate and toxicity of Prymnesium parvum and Prymnesium patelliferum (Haptophyta) in response to changes in salinity, light and temperature. Sarsia 83:409–418.

Ledig, F. T., and D. R. Korbobo. 1983. Adaptation of sugar maple populations along altitudinal gradients: photosynthesis, respiration, and specific leaf weight. American Journal of Botany pages 256–265.

Lee, K. L., and H. S. Kim. 2007. Growth characteristics of three synurophytes (Mallomonas species) at different temperatures and pH. Nova Hedwigia 84:227–240.

Lee, K. L., H.-S. Yoon, Y. J. Kim, and H. S. Kim. 2007. Growth characteristics of three Mallomonas species (silica-scaled chrysophytes) at different temperatures and pH. Nordic Journal of Botany 25:113–118.

Lehtovirta-Morley, L. E., C. Ge, J. Ross, H. Yao, G. W. Nicol, and J. I. Prosser. 2014. Characterisation of terrestrial acidophilic archaeal ammonia oxidisers and their inhibition and stimulation by organic compounds. FEMS Microbiol Ecol 89:542–552.

Lei, J., B. S. Payne, and S. Y. Wang. 1996. Filtration dynamics of the zebra mussel, Dreissena polymorpha . Can. J. Fish. Aquat. Sci. 53:29–37.

Leigh, J. A., F. Mayer, and R. S. Wolfe. 1981. Acetogenium kivui, a new thermophilic hydrogen-oxidizing acetogenic bacterium. Archives of Microbiology 129:275–280.

Lenz, M., R. A. Barrett, and E. R. Williams. 1982. Influence of diet on the survival and wood consumption of Porotermes adamsoni (Froggatt) (Isoptera: Termopsidae) at different temperatures. BER 72:423.

Lenz, M., and E. Williams. 1986. CHANGING FEEDING PATTERNS OF POROTERMES-ADAMSONI (FROGGATT)(ISOPTERA, TERMOPSIDAE) DURING NO-CHOICE AND CHOICE TRIALS AT DIFFERENT TEMPERATURES ON SOUND AND DECAYED NEST WOOD. Sociobiology 11:215–226.

Lenz, P. H., A. E. Hower, and D. K. Hartline. 2005. Temperature Compensation in the Escape Response of a Marine Copepod, Calanus finmarchicus (Crustacea). Biological Bulletin 209:75.

Leroi, F., P. A. Fall, M. F. Pilet, F. Chevalier, and R. Baron. 2012. Influence of temperature, pH and NaCl concentration on the maximal growth rate of Brochothrix thermosphacta and a bioprotective bacteria Lactococcus piscium CNCM I-4031. Food Microbiology 31:222–228.

Lester, W. W., M. S. Adams, and A. M. Farmer. 1988. Effects of light and temperature on photosynthesis of the nuisance alga Cladophora glomerata (L.) Kutz from Green Bay, Lake Michigan. New phytologist 109:53–58.

Leuschner, C., and U. Rees. 1993. CO 2 gas exchange of two intertidal seagrass species, Zostera marina L. and Zostera noltii Hornem., during emersion. Aquatic Botany 45:53–62.

Lewis, N., S. Bates, J. McLachlan, and J. Smith. 1993. Temperature effects on growth, domoic acid production, and morphology of the diatom Nitzschia pungens f. multiseries. Toxic phytoplankton blooms in the sea pages 601–606.

Li, D., G. Wang, L. Chen, F. Lü, and Z. Shen. 2009. Effects of Irradiance and Temperature on the Photosynthesis and Vegetative Propagation of Caulerpa serrulata. Journal of integrative plant biology 51:147–154.

Lieth, J., and C. Pasian. 1990. A model for net photosynthesis of rose leaves as a function of photosynthetically active radiation, leaf temperature, and leaf age. Journal of the American Society for Horticultural Science 115:486–491.

Lighton, J. R., G. A. Bartholomew, and D. H. Feener. 1987. Energetics of locomotion and load carriage and a model of the energy cost of foraging in the leaf-cutting ant Atta colombica Guer. Physiological Zoology pages 524–537.

Lighton, J. R. B., and F. D. Duncan. 2002. Energy Cost of Locomotion: Validation of Laboratory Data by in situ Respirometry. Ecology 83:3517.

Lindstrom, K. 1984. EFFECT OF TEMPERATURE, LIGHT AND pH ON GROWTH, PHOTOSYNTHESIS AND RESPIRATION OF THE DINOFLAGELLATE PERIDINIUM CINCTUM FA. WESTII IN LABORATORY CULTURES1. Journal of Phycology 20:212–220.

Linley, J. R. 1986. Swimming Behavior of the Larva of Culicoides Variipennis (Diptera: Ceratopogonidae) and its Relationship to Temperature and Viscosity. Journal of Medical Entomology 23:473–483.

Liu, X., and H. Ye. 2009. Effect of temperature on development and survival of Bactrocera correcta (Diptera: Tephritidae). Scientific Research and Essay 4:467–472.

Londos, P. L., and R. J. Brooks. 1988. Effect of temperature acclimation on locomotory performance curves in the toad, Bufo woodhousii woodhousii. Copeia pages 26–32.

Lovejoy, C., W. F. Vincent, S. Bonilla, S. Roy, M.-J. Martineau, R. Terrado, M. Potvin, R. Massana, and C. Pedrós-Alió. 2007. DISTRIBUTION, PHYLOGENY, AND GROWTH OF COLD-ADAPTED PICOPRASINOPHYTES IN ARCTIC SEAS. Journal of Phycology 43:78–89.

Ludlow, M., and G. Wilson. 1971. Photosynthesis of tropical pasture plants I. Illuminance, carbon dioxide concentration, leaf temperature, and leaf-air vapour pressure difference. Australian Journal of Biological Sciences 24:449–470.

Lundholm, N., J. Skov, R. Pocklington, and Ø. Moestrup. 1997. Studies on the marine planktonic diatom Pseudo-nitzschia. 2. Autecology of P. pseudodelicatissima based on isolates from Danish coastal waters. Phycologia 36:381–388.

LÜRLING, M., F. Eshetu, E. J. Faassen, S. Kosten, and V. L. Huszar. 2013. Comparison of cyanobacterial and green algal growth rates at different temperatures. Freshwater Biology 58:552–559.

MABERLY, S. C. 1985. Photosynthesis by fontinalis antipyretica. i. interaction between photon irradiance, concentration of carbon dioxide and temperature. New Phytologist 100:127–140.

Mackey, K. R., A. Paytan, K. Caldeira, A. R. Grossman, D. Moran, M. McIlvin, and M. A. Saito. 2013. Effect of temperature on photosynthesis and growth in marine Synechococcus spp. Plant physiology 163:815–829.

MADDUX, W. S., and R. F. JONES. 1964. Some interactions of temperature,light intensity, and nutrient concentration during the continuous culture of Nitzschia closterium and Tetraselmis sp. Limnol. Oceangr. 9:79–86.

Marsh, A. C. 1985. Microclimatic factors influencing foraging patterns and success of the thermophilic desert ant,Ocymyrmex barbiger. Insectes Sociaux 32:286–296.

Marsh, J. A., W. C. Dennison, and R. S. Alberte. 1986. Effects of temperature on photosynthesis and respiration in eelgrass (Zostera marina L.). Journal of Experimental Marine Biology and Ecology 101:257–267.

Marsh, R. L., and A. F. Bennett. 1986. Thermal dependence of sprint performance of the lizard Sceloporus occidentalis. Journal of Experimental Biology 126:79–87.

Marvin, G. A. 2003*a*. Aquatic and Terrestrial Locomotor Performance in a Semiaquatic Plethodontid Salamander (Pseudotriton ruber): Influence of Acute Temperature, Thermal Acclimation, and Body Size. Copeia 2003:704–713.

———. 2003*b*. Effects of acute temperature and thermal acclimation on aquatic and terrestrial locomotor performance of the three-lined salamander, Eurycea guttolineata. Journal of Thermal Biology 28:251–259.

Mathieson, A. C., and R. L. Burns. 1971. Ecological studies of economic red algae. I. Photosynthesis and respiration of Chondrus crispus Stackhouse and Gigartina stellata (Stackhouse) Batters. Journal of Experimental Marine Biology and Ecology 7:197–206.

Mathieson, A. C., and C. J. Dawes. 1974. Ecological studies of Floridian Eucheuma (Rhodophyta, Gigartinales). II. Photosynthesis and respiration. Bulletin of Marine Science 24:274–285.

Mayo, A. W. 1997. Effects of temperature and pH on the kinetic growth of unialga Chlorella vulgaris cultures containing bacteria. Water environment research pages 64–72.

McCaffrey, J. P., and R. L. Horsburgh. 1986. Functional Response of Orius insidiosus (Hemiptera: Anthocoridae) to the European Red Mite, Panonychus ulmi (Acari: Tetranychidae), at Different Constant Temperatures. Environmental Entomology 15:532–535.

McCombie, A. M. 1960. Actions and interactions of temperature, light intensity and nutrient concentration on the growth of the green alga, Chlamydomonas reinhardi Dangeard. Journal of the Fisheries Board of Canada 17:871–894.

McCoull, C., R. Swain, and R. Barnes. 1998. Effect of temperature on the functional response and components of attack rate inNaucoris congrexStål (Hemiptera: Naucoridae). Australian Journal of Entomology 37:323–327.

McKay, L., D. Kamykowski, E. Milligan, B. Schaeffer, and G. Sinclair. 2006. Comparison of swimming speed and photophysiological responses to different external conditions among three Karenia brevis strains. Harmful Algae 5:623–636.

McLeese, D., and D. Wilder. 1958. The activity and catchability of the lobster (Homarus americanus) in relation to temperature. Journal of the Fisheries Board of Canada 15:1345–1354.

McMeekin, T. A., and P. D. Franzmann. 1988. Effect of temperature on the growth rates of halotolerant and halophilic bacteria isolated from Antarctic saline lakes. Polar Biol 8:281–285.

Meade, M. E., J. E. Doeller, D. Kraus, and S. A. Watts. 2002. Effects of Temperature and Salinity on Weight Gain, Oxygen Consumption Rate, and Growth Efficiency in Juvenile Red-Claw Crayfish Cherax quadricarinatus . Journal of the World Aquaculture Society 33:188–198.

Mehnert, G., F. Leunert, S. Cirés, K. D. Jöhnk, J. Rücker, B. Nixdorf, and C. Wiedner. 2010. Competitiveness of invasive and native cyanobacteria from temperate freshwaters under various light and temperature conditions. Journal of Plankton Research 32:1009–1021.

Meier, K. S. 2004. Effect of temperature on culture growth and cyst production in the calcareous dinoflagellates Calciodinellum albatrosianum, Leonella granifera and Pernambugia tuberosa. Micropaleontology 50:93–106.

MEMBRE, J., B. LEPORQ, M. VIALETTE, E. METTLER, L. PERRIER, D. THUAULT, and M. ZWIETERING. 2005. Temperature effect on bacterial growth rate: quantitative microbiology approach including cardinal values and variability estimates to perform growth simulations on/in food. International Journal of Food Microbiology 100:179–186.

Menon, A., P. W. Flinn, and B. A. Dover. 2002. Influence of temperature on the functional response of Anisopteromalus calandrae (Hymenoptera: Pteromalidae), a parasitoid of Rhyzopertha dominica (Coleoptera: Bostrichidae). Journal of Stored Products Research 38:463–469.

Messenger, P., and D. Force. 1963. An Experimental Host-Parasite System: Therioaphis maculata (Buckton)-Praon palitans Muesebeck (Homoptera: Aphididae–Hymenoptera: Braconidae). Ecology 44:532–540.

Messenger, P. S. 1968. BIOCLIMATIC STUDIES OF THE APHID PARASITE PRAON EXSOLETUM: I. EFFECTS OF TEMPERATURE ON THE FUNCTIONAL RESPONSE OF FEMALES TO VARYING HOST DENSITIES. The Canadian Entomologist 100:728–741.

Miller, K. 1982. Effect of Temperature on Sprint Performance in the Frog Xenopus laevis and the Salamander Necturus maculosus. Copeia 1982:695.

Miller, R. L., and D. L. Kamykowski. 1986. EFFECTS OF TEMPERATURE, SALINITY, IRRADIANCE AND DIURNAL PERIODICITY ON GROWTH AND PHOTOSYNTHESIS IN THE DIATOM NITZSCHIA AMERICANA; LIGHT-SATURATED GROWTH. Journal of Phycology 22:339–348.

Mitrovic, S. M., J. N. Hitchcock, A. W. Davie, and D. A. Ryan. 2010. Growth responses of Cyclotella meneghiniana (Bacillariophyceae) to various temperatures. Journal of plankton research 32:1217–1221.

Miyamoto, K., P. C. Hallenbeck, and J. R. Benemann. 1979. Nitrogen fixation by thermophilic blue-green algae (cyanobacteria): temperature characteristics and potential use in biophotolysis. Applied and environmental microbiology 37:454–458.

Mjaaland, G. 1956. Some Laboratory Experiments on the Coccolithophorid Coccolithus huxleyi. Oikos 7:251.

Mohaghegh, D. Clercq, and Tirry. 2001. Functional response of the predators Podisus maculiventris (Say) and Podisus nigrispinus (Dallas) (Het., Pentatomidae) to the beet armyworm, Spodoptera exigua (Hubner) (Lep., Noctuidae): ef fect of temperature. J Appl Entomology 125:131–134.

Monson, R. K., C. H. Jaeger, W. W. Adams, E. M. Driggers, G. M. Silver, and R. Fall. 1992. Relationships among Isoprene Emission Rate, Photosynthesis, and Isoprene Synthase Activity as Influenced by Temperature. PLANT PHYSIOLOGY 98:1175–1180.

Monson, R. K., R. O. Littlejohn Jr, and G. J. Williams III. 1983. Photosynthetic adaptation to temperature in four species from the Colorado shortgrass steppe: a physiological model for coexistence. Oecologia 58:43–51.

Monson, R. K., M. A. Stidham, G. J. Williams, G. E. Edwards, and E. G. Uribe. 1982. Temperature Dependence of Photosynthesis in Agropyron smithii Rydb. : I. FACTORS AFFECTING NET CO2 UPTAKE IN INTACT LEAVES AND CONTRIBUTION FROM RIBULOSE-1,5-BISPHOSPHATE CARBOXYLASE MEASURED IN VIVO AND IN VITRO. PLANT PHYSIOLOGY 69:921–928.

Montagnes, D. J., and M. Franklin. 2001. Effect of temperature on diatom volume, growth rate, and carbon and nitrogen content: reconsidering some paradigms. Limnology and Oceanography 46:2008–2018.

Montresor, M., and C. R. Tomas. 1988. GROWTH AND PROBABLE GAMETE FORMATION IN THE MARINE DINOFLAGELLATE CERATIUM SCHRANKII. Journal of Phycology 24:495–502.

Mooney, H., and M. West. 1964. Photosynthetic acclimation of plants of diverse origin. American Journal of Botany pages 825–827.

Mooney, H. A. 1980. Photosynthetic plasticity of populations of Heliotropium curassavicum L. originating from differing thermal regimes. Oecologia 45:372–376.

Mooney, H. A., O. Björkman, and G. J. Collatz. 1978. Photosynthetic acclimation to temperature in the desert shrub, Larrea divaricata I. Carbon dioxide exchange characteristics of intact leaves. Plant Physiology 61:406–410.

Moore, L., R. Goericke, and S. Chisholm. 1995. Comparative physiology of Synechococcus and Prochlorococcus: influence of light and temperature on growth, pigments, fluorescence and absorptive properties. Marine Ecology Progress Series 116:259–275.

Morehead, S. A., and D. H. F. Jr. 1998. Foraging behavior and morphology: seed selection in the harvester ant genus, Pogonomyrmex. Oecologia 114:548–555.

Morris, E. P., and J. C. Kromkamp. 2003. Influence of temperature on the relationship between oxygen- and fluorescence-based estimates of photosynthetic parameters in a marine benthic diatom (Cylindrotheca closterium) . European Journal of Phycology 38:133–142.

Mortensen, S. H., K. Y. Børsheim, J. Rainuzzo, and G. Knutsen. 1988. Fatty acid and elemental composition of the marine diatom Chaetoceros gracilis Schütt. Effects of silicate deprivation, temperature and light intensity. Journal of Experimental Marine Biology and Ecology 122:173–185.

Morton, S. L., D. R. Norris, and J. W. Bomber. 1992. Effect of temperature, salinity and light intensity on the growth and seasonality of toxic dinoflagellates associated with ciguatera. Journal of experimental marine biology and ecology 157:79–90.

Moss, B. 1973. The Influence of Environmental Factors on the Distribution of Freshwater Algae: An Experimental Study: III. Effects of Temperature, Vitamin Requirements and Inorganic Nitrogen Compounds on Growth. The Journal of Ecology pages 179–192.

Mtimet, N., C. Trunet, A.-G. Mathot, L. Venaille, I. Leguérinel, L. Coroller, and O. Couvert. 2015. Modeling the behavior of Geobacillus stearothermophilus ATCC 12980 throughout its life cycle as vegetative cells or spores using growth boundaries. Food Microbiology 48:153–162.

Muller, H. v. 1972. Wachstum und Phosphat-bedurf von Nitzschia actinastroides (Lemn.) V. Goor. in statischer und homokontinuierlicher Kultur unter Phosphat-limitierung. Arch. Hydrobiol pages 399–484.

Myking, T. 1997. Effects of constant and fluctuating temperature on time to budburst in Betula pubescens and its relation to bud respiration. Trees 12:107–112.

Nadgauda, D., and H. N. Pitre. 1986. Effects of Temperature on Feeding, Development, Fecundity, and Longevity of Nabis roseipennis (Hemiptera: Nabidae) Fed Tobacco Budworm (Lepidoptera: Noctuidae) Larvae and Tarnished Plant Bug (Hemiptera: Miridae) Nymphs. Environmental Entomology 15:536–539.

Nagasoe, S., D.-I. Kim, Y. Shimasaki, Y. Oshima, M. Yamaguchi, and T. Honjo. 2006. Effects of temperature, salinity and irradiance on the growth of the red tide dinoflagellate Gyrodinium instriatum Freudenthal et Lee. Harmful algae 5:20–25.

Nakamura, Y., and M. M. Watanabe. 1983. Growth characteristics ofChattonella antiqua (Raphidophyceae). Journal of the Oceanographical Society of Japan 39:110–114.

Nalewajko, C., and T. P. Murphy. 2001. Effects of temperature, and availability of nitrogen and phosphorus on the abundance of Anabaena and Microcystis in Lake Biwa, Japan: an experimental approach. Limnology 2:45–48.

Navarro, J., G. Leiva, G. Martinez, and C. Aguilera. 2000. Interactive effects of diet and temperature on the scope for growth of the scallop Argopecten purpuratus during reproductive conditioning. Journal of Experimental Marine Biology and Ecology 247:67–83.

Navas, C. A. 1996. Metabolic Physiology, Locomotor Performance, and Thermal Niche Breadth in Neotropical Anurans. Physiological Zoology 69:1481–1501.

Nedwell, D., and M. Rutter. 1994. Influence of temperature on growth rate and competition between two psychrotolerant Antarctic bacteria: low temperature diminishes affinity for substrate uptake. Applied and Environmental Microbiology 60:1984–1992.

Neilson, R. E., M. M. Ludlow, and P. G. Jarvis. 1972. Photosynthesis in Sitka Spruce (Picea sitchensis (Bong.) Carr.). II. response to temperature. The Journal of Applied Ecology 9:721.

Nevot, M., V. Deroncelé, M. J. Montes, and E. Mercade. 2008. Effect of incubation temperature on growth parameters of Pseudoalteromonas antarctica NF 3 and its production of extracellular polymeric substances . Journal of Applied Microbiology 105:255–263.

Newell, R., and V. Pye. 1968. Seasonal variations in the effect of temperature on the respiration of certain intertidal algae. Journal of the Marine Biological Association of the United Kingdom 48:341–348.

Newell, R. C., L. G. Johson, and L. H. Kofoed. 1977. Adjustment of the components of energy balance in response to temperature change in Ostrea edulis. Oecologia 30:97–110.

Nielsen, M. 1996. Growth and chemical composition of the toxic dinoflagellate Gymnodinium galatheanum in relation to irradiance, temperature and salinity. Marine Ecology Progress Series 136:205–211.

Nielsen, M. V., and C. P. Tønseth. 1991. Temperature and salinity effect on growth and chemical composition of Gyrodinium aureolum Hulburt in culture . J Plankton Res 13:389–398.

Nishibori, N., T. Nishijima, Y. Onoda, and Y. Hata. 1991. Effects of light intensity, temperature, pH, and nitrogenous nutrient on the growth of Peridinium bipes fo. occultatum [in the lakes, Japan]. Bulletin of the Japanese Society of Scientific Fisheries (Japan) .

Nishikawa, T., and M. Yamaguchi. 2006. Effect of temperature on light-limited growth of the harmful diatom Eucampia zodiacus Ehrenberg, a causative organism in the discoloration of Porphyra thalli. Harmful Algae 5:141–147.

Niu, C., D. Lee, S. Goshima, and S. Nakao. 2003. Effects of temperature on food consumption, growth and oxygen consumption of freshwater prawn Macrobrachium rosenbergii (de Man 1879) postlarvae. Aquaculture Research 34:501–506.

Nordli, E. 1957. Experimental Studies on the Ecology of Ceratia. Oikos 8:200.

Nozhevnikova, A., M. Simankova, S. Parshina, and O. Kotsyurbenko. 2001. Temperature characteristics of methanogenic archaea and acetogenic bacteria isolated from cold environments. Water science and technology 44:41–48.

Oertli, J. 1989. Relationship of wing beat frequency and temperature during take-off flight in temperate-zone beetles. Journal of experimental biology 145:321–338.

Ohkubo, N., O. Yagi, and M. Okada. 1991. Effects of temperature and illumination on the growth of blue-green alga microcystis viridis. Japanese Journal of Limnology (Rikusuigaku Zasshi) 52:255–261.

Ojala, A. 1993. EFFECTS OF TEMPERATURE AND IRRADIANCE ON THE GROWTH OF TWO FRESHWATER PHOTOSYNTHET1C CRYPTOPHYTES. Journal of phycology 29:278–284.

Ono, K., S. Khan, and Y. Onoue. 2000. Effects of temperature and light intensity on the growth and toxicity of Heterosigma akashiwo (Raphidophyceae). Aquaculture Research 31:427–433.

Orr, P. T., J. Pokornỳ, P. Denny, and P. J. Sale. 1988. Photosynthetic response of Myriophyllum salsugineum AE Orchard to photon irradiance, temperature and external free CO 2. Aquatic botany 30:363–378.

Oshiki, M., M. Shimokawa, N. Fujii, H. Satoh, and S. Okabe. 2011. Physiological characteristics of the anaerobic ammonium-oxidizing bacterium Candidatus Brocadia sinica. Microbiology 157:1706–1713.

O'Steen, S., and A. F. Bennett. 2003. Thermal acclimation effects differ between voluntary, maximum, and critical swimming velocities in two cyprinid fishes. Physiological and Biochemical Zoology 76:484–496.

Paarup, M., M. W. Friedrich, B. J. Tindall, and K. Finster. 2005. Characterization of the psychrotolerant acetogen strain SyrA5 and the emended description of the species Acetobacterium carbinolicum. Antonie Van Leeuwenhoek 89:55–69.

Paasche, E. 1967. Marine Plankton Algae Grown with Light-Dark Cycles. I. Coccolithus huxleyi. Physiologia Plantarum 20:946–956.

———. 1968. Marine Plankton Algae Grown with Light-Dark Cycles. Physiologia plantarum 21:66–77.

Padilla-Gamino, J. L., and R. C. Carpenter. 2007. Seasonal acclimatization of Asparagopsis taxiformis (Rhodophyta) from different biogeographic regions. Limnology and oceanography 52:833–842.

PALTA, J. A., and P. S. NOBEL. 1989*a*. Influences of water status, temperature, and root age on daily patterns of root respiration for two cactus species. Annals of Botany 63:651–662.

———. 1989*b*. Root respiration for Agave deserti: influence of temperature, water status and root age on daily patterns. Journal of Experimental Botany 40:181–186.

Patel, B. K. C., C. Monk, H. Littleworth, H. W. Morgan, and R. M. Daniel. 1987. Clostridium fervidus sp. nov., a New Chemoorganotrophic Acetogenic Thermophile. International Journal of Systematic Bacteriology 37:123–126.

Patterson, D. T., and D. A. Mortensen. 1985. Effects of temperature and photoperiod on common crupina (Crupina vulgaris). Weed Science pages 333–339.

Paul, M., D. Lawlor, and S. P. DRISCOLL. 1990. The effect of temperature on photosynthesis and carbon fluxes in sunflower and rape. Journal of Experimental Botany 41:547–555.

Pearcy, R., A. Harrison, H. Mooney, and O. Björkman. 1974. Seasonal changes in net photosynthesis of Atriplex hymenelytra shrubs growing in Death Valley, California. Oecologia 17:111–121.

Pearcy, R. W. 1977. Acclimation of Photosynthetic and Respiratory Carbon Dioxide Exchange to Growth Temperature in Atriplex lentiformis (Torr.) Wats. PLANT PHYSIOLOGY 59:795–799.

Pérez, M., and J. Romero. 1992. Photosynthetic response to light and temperature of the seagrass Cymodocea nodosa and the prediction of its seasonality. Aquatic Botany 43:51–62.

Pérez-Lloréns, J., and F. X. Niell. 1993. Temperature and emergence effects on the net photosynthesis of two Zostera noltii Hornem. morphotypes. Hydrobiologia 254:53–64.

PERSSON, L. 1979. The effects of temperature and different food organisms on the rate of gastric evacuation in perch (Perca fluviatilis). Freshwater Biology 9:99–104.

Persson, L. 1986. Temperature-Induced Shift in Foraging Ability in Two Fish Species, Roach (Rutilus rutilus) and Perch (Perca fluviatilis): Implications for Coexistence between Poikilotherms. The Journal of Animal Ecology 55:829.

Peterson, R. H., and J. M. Anderson. 1969. Influence of Temperature Change on Spontaneous Locomotor Activity and Oxygen Consumption of Atlantic Salmon, Salmo salar , Acclimated to Two Temperatures . J. Fish. Res. Bd. Can. 26:93–109.

Petron, G., P. Harley, J. Greenberg, and A. Guenther. 2001. Seasonal temperature variations influence isoprene emission. Geophysical Research Letters 28:1707–1710.

Pilon, J., and L. Santamaria. 2001. Seasonal acclimation in the photosynthetic and respiratory temperature responses of three submerged freshwater macrophyte species. New Phytologist 151:659–670.

Pittermann, J., and R. F. Sage. 2001. The response of the high altitude C4 grass Muhlenbergia montana (Nutt.) AS Hitchc. to long-and short-term chilling. Journal of Experimental Botany 52:829–838.

Podolsky, R., and R. Emlet. 1993. Separating the effects of temperature and viscosity on swimming and water movement by sand dollar larvae (Dendraster excentricus). The Journal of Experimental Biology 176:207–222.

Post, A. F., R. de Wit, and L. R. Mur. 1985. Interactions between temperature and light intensity on growth and photosynthesis of the cyanobacterium Oscillatoria agardhii. Journal of Plankton Research 7:487–495.

Putnam, R. W., and A. F. Bennett. 1981. Thermal dependence of behavioural performance of anuran amphibians. Animal Behaviour 29:502–509.

Qin, J. G., and Y. Li. 2006. Optimization of the growth environment of Botryococcus braunii strain CHN 357. Journal of Freshwater Ecology 21:169–176.

RAFIQUL, I. M., A. Hassan, G. Sulebele, C. Orosco, and P. Roustaian. 2003. Influence of temperature on growth and biochemical composition of Spirulina platensis and S. fusiformis .

Raimbault, P. 1984. Influence of temperature on the transient response in nitrate uptake and reduction by four marine diatoms. Journal of experimental marine biology and ecology 84:37–53.

Rand, A. S. 1964. Inverse Relationship Between Temperature and Shyness in the Lizard Anolis Lineatopus. Ecology 45:863–864.

Rao, K. P. 1953. Rate of Water Propulsion in Mytilus Californianus as a Function of Latitude. Biological Bulletin 104:171.

Rapala, J., and K. Sivonen. 1998. Assessment of environmental conditions that favor hepatotoxic and neurotoxic Anabaena spp. strains cultured under light limitation at different temperatures. Microbial Ecology 36:181–192.

Rastorfer, J. R. 1970. Effects of Light Intensity and Temperature on Photosynthesis and Respiration of Two East Antarctic Mosses, Bryum argenteum and Bryum antarcticum. The Bryologist 73:544.

Ratkowsky, D. A., J. Olley, and T. Ross. 2005. Unifying temperature effects on the growth rate of bacteria and the stability of globular proteins. Journal of Theoretical Biology 233:351–362.

Read, J. 1990. Some Effects of Acclimation Temperature on Net Photosynthesis in Some Tropical and Extra-Tropical Australasian Nothofagus Species. The Journal of Ecology 78:100.

Reay, D. S., D. B. Nedwell, J. Priddle, and J. C. Ellis-Evans. 1999. Temperature dependence of inorganic nitrogen uptake: reduced affinity for nitrate at suboptimal temperatures in both algae and bacteria. Applied and Environmental Microbiology 65:2577–2584.

Regehr, D. L., and F. Bazzaz. 1976. Low temperature photosynthesis in successional winter annuals. Ecology pages 1297–1303.

Reicosky, D., P. Brown, and M. Moran. 1994. Diurnal trends in wheat canopy temperature, photosynthesis, and evapotranspiration. Remote Sensing of Environment 49:235–245.

Renaud, J. M., and E. D. Stevens. 1983. The extent of long-term temperature compensation for jumping distance in the frog, Rana pipiens , and the toad, Bufo americanus . Can. J. Zool. 61:1284–1287.

Renaud, S. M., L.-V. Thinh, G. Lambrinidis, and D. L. Parry. 2002. Effect of temperature on growth, chemical composition and fatty acid composition of tropical Australian microalgae grown in batch cultures. Aquaculture 211:195–214.

Renaud, S. M., H. C. Zhou, D. L. Parry, L.-V. Thinh, and K. C. Woo. 1995. Effect of temperature on the growth, total lipid content and fatty acid composition of recently isolated tropical microalgae Isochrysis sp., Nitzschia closterium, Nitzschia paleacea, and commercial species Isochrysis sp. (clone T.ISO). J Appl Phycol 7:595–602.

Rengefors, K., G. A. Weyhenmeyer, and I. Bloch. 2012. Temperature as a driver for the expansion of the microalga Gonyostomum semen in Swedish lakes. Harmful algae 18:65–73.

Reynolds, W. W., and M. E. Casterlin. 1979. Behavioral thermoregulation and activity in Homarus americanus. Comparative Biochemistry and Physiology Part A: Physiology 64:25–28.

Rhee, G., I. J. Gotham, et al. 1981. The effect of environmental factors on phytoplankton growth: temperature and the interactions of temperature with nutrient limitation. Limnology and Oceanography 26:635–648.

Rhodes, L. L., B. M. Peake, A. L. MacKenzie, and S. Marwick. 1995. Coccolithophores Gephyrocapsa oceanica and Emiliania huxleyi (Prymnesiophyceae= Haptophyceae) in New Zealand's coastal waters: characteristics of blooms and growth in laboratory culture. New Zealand Journal of Marine and Freshwater Research 29:345–357.

Richards, S., and C. Bull. 1990. Size-limited predation on tadpoles of three Australian frogs. Copeia pages 1041–1046.

Rigatuso, R., S. M. R. Bertoluzzo, F. E. Quattrin, and M. G. Bertoluzzo. 2000. Ant Activity Associated with a Chemical Compound. J. Chem. Educ. 77:183.

Riisgård, H. U., and D. F. Seerup. 2003. Filtration rates in the soft clam Mya arenaria: effects of temperature and body size. Sarsia 88:416–428.

Rissing, S. W. 1982. Foraging Velocity of Seed-Harvester Ants, Veromessor pergandei (Hymenoptera: Formicidae). Environmental Entomology 11:905–907.

Robakowski, P., P. Montpied, and E. Dreyer. 2002. Temperature response of photosynthesis of silver fir (Abies alba Mill.) seedlings . Ann. For. Sci. 59:163–170.

Robichaux, R. H., and R. W. Pearcy. 1980. Photosynthetic responses of C3 and C4 species from cool shaded habitats in Hawaii. Oecologia 47:106–109.

Robinson, J. L., B. Pyzyna, R. G. Atrasz, C. A. Henderson, K. L. Morrill, A. M. Burd, E. DeSoucy, R. E. Fogleman, J. B. Naylor, S. M. Steele, D. R. Elliott, K. J. Leyva, and R. F. Shand. 2005. Growth Kinetics of Extremely Halophilic Archaea (Family Halobacteriaceae) as Revealed by Arrhenius Plots. Journal of Bacteriology 187:923–929.

Ruberson, J., C. Tauber, and M. Tauber. 1995. Developmental Effects of Host and Temperature on Telenomus spp (Hymenoptera: Scelionidae) Parasitizing Chrysopid Eggs. Biological Control 5:245–250.

Runjie, Z., K. L. Heong, and I. T. Domingo. 1996. Relationship Between Temperature and Functional Response in Cardiochiles philippinensis (Hymenoptera: Braconidae), a Larval Parasitoid of Cnaphalocrocis medinalis (Lepidoptera: Pyralidae). Environmental Entomology 25:1321–1324.

Ruyet, J. P.-L., K. Mahé, N. L. Bayon, and H. L. Delliou. 2004. Effects of temperature on growth and metabolism in a Mediterranean population of European sea bass, Dicentrarchus labrax. Aquaculture 237:269–280.

Ryther, J. H. 1954. The Ecology of Phytoplankton Blooms in Moriches Bay and Great South Bay, Long Island, New York. Biological Bulletin 106:198.

Sabour, B., B. Sbiyyaa, M. Loudiki, B. Oudra, M. Belkoura, and V. Vasconcelos. 2009. Effect of light and temperature on the population dynamics of two toxic bloom forming Cyanobacteria–Microcystis ichthyoblabe and Anabaena aphanizomenoides. Chemistry and Ecology 25:277–284.

Sage, R. F., and T. D. Sharkey. 1987. The effect of temperature on the occurrence of O2 and CO2 insensitive photosynthesis in field grown plants. Plant Physiology 84:658–664.

Saker, M. L., and G. K. Eaglesham. 1999. The accumulation of cylindrospermopsin from the cyanobacterium Cylindrospermopsis raciborskii in tissues of the Redclaw crayfish Cherax quadricarinatus. Toxicon 37:1065–1077.

Salvucci, M. E., and S. J. Crafts-Brandner. 2004. Relationship between the heat tolerance of photosynthesis and the thermal stability of Rubisco activase in plants from contrasting thermal environments. Plant Physiology 134:1460–1470.

Sánchez, J., J. Fernández-Sevilla, F. Acién, M. Cerón, J. Pérez-Parra, and E. Molina-Grima. 2008. Biomass and lutein productivity of Scenedesmus almeriensis: influence of irradiance, dilution rate and temperature. Applied microbiology and biotechnology 79:719–729.

Sanchez-Salazar, M., C. Griffiths, and R. Seed. 1987. The effect of size and temperature on the predation of cockles Cerastoderma edule (L.) by the shore crab Carcinus maenas (L.). Journal of Experimental Marine Biology and Ecology 111:181–193.

Sandnes, J., T. Källqvist, D. Wenner, and H. R. Gislerød. 2005. Combined influence of light and temperature on growth rates of Nannochloropsis oceanica: linking cellular responses to large-scale biomass production. Journal of Applied Phycology 17:515–525.

Schöne, H. K. 1982. The influence of light and temperature on the growth rates of six phytoplankton species from the upwelling area off northwest africa. Rapp. Pv. Reun. Cons. Int. Explor. Mer 189:246–253.

Scribner, S. J., and P. J. Weatherhead. 1995. Locomotion and antipredator behaviour in three species of semi-aquatic snakes. Can. J. Zool. 73:321–329.

Seaburg, K. G., B. C. Parked, R. A. Wharton, and G. M. Simmons. 1981. TEMPERATURE-GROWTH RESPONSES OF ALGAL ISOLATES FROM ANTARCTIC OASES. Journal of Phycology 17:353–360.

Seemann, J. R., J. A. Berry, and W. J. S. Downton. 1984. Photosynthetic Response and Adaptation to High Temperature in Desert Plants A Comparison of Gas Exchange and Fluorescence Methods for Studies of Thermal Tolerance. Plant Physiology 75:364–368.

Seemann, J. R., W. J. S. Downton, and J. A. Berry. 1986. Temperature and leaf osmotic potential as factors in the acclimation of photosynthesis to high temperature in desert plants. Plant Physiology 80:926–930.

Seguel, M. R. 1992. Interactive effects of temperature-light and temperature-salinity on growth of five phytoplanktonic species isolated from a shallow-water embayment of Nova Scotia.

Seki, H., H. Ozawa, and S.-e. Ichimura. 1981. Temperature dependence of filament length of Anabaena spiroides Klebahn var. crassa Lemm. Hydrobiologia 83:419–423.

Senft, W. H., R. A. Hunchberger, and K. E. Roberts. 1981. TEMPERATURE DEPENDENCE OF GROWTH AND PHOSPHORUS UPTAKE IN TWO SPECIES OF VOLVOX (VOLVOCALES, CHLOROPHYTA). Journal of Phycology 17:323–329.

Seymour, R. S., and P. Schultze-Motelf. 1999. Respiration, temperature regulation and energetics of thermogenic inflorescences of the dragon lily Dracunculus vulgaris (Araceae). Proceedings of the Royal Society B: Biological Sciences 266:1975–1983.

Shapley, H. 1920. Thermokinetics of Liometopum Apiculatum Mayr. Proceedings of the National Academy of Sciences 6:204–211.

———. 1924. Note on the Thermokinetics of Dolichoderine Ants. Proceedings of the National Academy of Sciences 10:436–439.

Sharkey, T. D., and F. Loreto. 1993. Water stress, temperature, and light effects on the capacity for isoprene emission and photosynthesis of kudzu leaves. Oecologia 95:328–333.

Simankova, M. V., O. R. Kotsyurbenko, E. Stackebrandt, N. A. Kostrikina, A. M. Lysenko, G. A. Osipov, and A. N. Nozhevnikova. 2000. Acetobacterium tundrae sp. nov., a new psychrophilic acetogenic bacterium from tundra soil. Archives of Microbiology 174:440–447.

Simpson, P., and J. Eaton. 1986. Comparative studies of the photosynthesis of the submerged macrophyte Elodea canadensis and the filamentous algae Cladophora glomerata and Spirogyra sp. Aquatic Botany 24:1–12.

Sinsawat, V., J. Leipner, P. Stamp, and Y. Fracheboud. 2004. Effect of heat stress on the photosynthetic apparatus in maize (Zea mays L.) grown at control or high temperature. Environmental and Experimental Botany 52:123–129.

Skillman, J. B., B. R. Strain, and C. B. Osmond. 1996. Contrasting patterns of photosynthetic acclimation and photoinhibition in two evergreen herbs from a winter deciduous forest. Oecologia 107:446–455.

Smayda, T. J. 1969. EXPERIMENTAL OBSERVATIONS ON THE INFLUENCE OF TEMPERATURE, LIGHT, AND SALINITY ON CELL DIVISION OF THE MARINE DIATOM, DETONULA CONFERVACEA (CLEVE) GRAN2. Journal of Phycology 5:150–157.

Smith, C. M., and J. A. Berry. 1986. Recovery of photosynthesis after exposure of intertidal algae to osmotic and temperature stresses: comparative studies of species with differing distributional limits. Oecologia 70:6–12.

Smith, D. G. 1997. Ecological factors influencing the antipredator behaviors of the ground skink, Scincella lateralis . Behavioral Ecology 8:622–629.

Smith, E. M., and E. B. Hadley. 1974. Photosynthetic and respiratory acclimation to temperature in Ledum groenlandicum populations. Arctic and Alpine Research pages 13–27.

Smith, G. R., and J. A. Lemos-Espinal. 2005. COMPARATIVE ESCAPE BEHAVIOR OF FOUR SPECIES OF MEXICAN PHRYNOSOMATID LIZARDS. Herpetologica 61:225–232.

Smith, L. 1994. Temperature Influences Functional Response of Anisopteromalus calandrae (Hymenoptera: Pteromalidae) Parasitizing Maize Weevil Larvae in Shelled Corn. Annals of the Entomological Society of America 87:849–855.

Smith, R. E., L. C. Stapleford, and R. S. Ridings. 1994. THE ACCLIMATED RESPONSE OF GROWTH, PHOTOSYNTHESIS, COMPOSITION, AND CARBON BALANCE TO TEMPERATURE IN THE PSYCHROPHILIC ICE DIATOM NITZSCHIA SERIATA. Journal of phycology 30:8–16.

Sorokin, C. 1960. Kinetic studies of temperature effects on the cellular level. Biochimica et biophysica acta 38:197–204.

Sosik, H. M., and B. G. Mitchell. 1994. EFFECTS OF TEMPERATURE ON GROWTH, LIGHT ABSORPTION, AND QUANTUM YIELD IN DUNALIELLA TERTIOLECTA (CHLOROPHYCEAE). Journal of Phycology 30:833–840.

Spencer, W. E., and R. G. Wetzel. 1993. Acclimation of photosynthesis and dark respiration of a submersed angiosperm beneath ice in a temperate lake. Plant physiology 101:985–991.

Staehr, P. A., and M. J. Birkeland. 2006. Temperature acclimation of growth, photosynthesis and respiration in two mesophilic phytoplankton species. Phycologia 45:648–656.

Stamenković, M., and D. Hanelt. 2013. Adaptation of growth and photosynthesis to certain temperature regimes is an indicator for the geographical distribution of Cosmarium strains (Zygnematophyceae, Streptophyta). European Journal of phycology 48:116–127.

Stapleford, L. S., and R. E. Smith. 1996. The interactive effects of temperature and silicon limitation on the psychrophilic ice diatom Pseudonitszchia seriata. Polar Biology 16:589–594.

Steigenberger, L. W., and P. A. Larkin. 1974. Feeding Activity and Rates of Digestion of Northern Squawfish (Ptychocheilus oregonensis) . J. Fish. Res. Bd. Can. 31:411–420.

Steller, D., J. Hernández-Ayón, R. Riosmena-Rodriguez, and A. Cabello-Pasini. 2007. Effect of temperature on photosynthesis, growth and calcification rates of the free-living coralline alga Lithophyllum margaritae. Ciencias Marinas 33:441–456.

Stevenson, R., and R. K. JOSEPHSON. 1990. Effects of operating frequency and temperature on mechanical power output from moth flight muscle. Journal of Experimental Biology 149:61–78.

Stevenson, R. D., C. R. Peterson, and J. S. Tsuji. 1985. The thermal dependence of locomotion, tongue flicking, digestion, and oxygen consumption in the wandering garter snake. Physiological Zoology pages 46–57.

Strzepek, R., and N. Price. 2000. Influence of irradiance and temperature on the iron content of the marine diatom Thalassiosira weissflogii (Bacillariophyceae). Marine Ecology Progress Series 206:107–117.

Sundström, A. M., A. Kremp, N. Daugbjerg, Ø. Moestrup, M. Ellegaard, R. Hansen, and S. Hajdu. 2009. GYMNODINIUM COROLLARIUM SP. NOV.(DINOPHYCEAE)A NEW COLD-WATER DINOFLAGELLATE RESPONSIBLE FOR CYST SEDIMENTATION EVENTS IN THE BALTIC SEA. Journal of Phycology 45:938–952.

Suzuki, Y., and M. Takahashi. 1995. GROWTH RESPONSES OF SEVERAL DIATOM SPECIES ISOLATED FROM VARIOUS ENVIRONMENTS TO TEMPERATURE. Journal of Phycology 31:880–888.

Swarup, A., J. Lu, K. C. DeWoody, and M. R. Antoniewicz. 2014. Metabolic network reconstruction, growth characterization and 13C-metabolic flux analysis of the extremophile Thermus thermophilus HB8. Metabolic Engineering 24:173–180.

Takahashi, M., and S.-e. Ichimura. 1970. Photosynthetic properties and growth of photosynthetic sulfur bacteria in lakes. Limnol. Oceanogr 15:929–944.

Takai, K., K. Nakamura, T. Toki, U. Tsunogai, M. Miyazaki, J. Miyazaki, H. Hirayama, S. Nakagawa, T. Nunoura, and K. Horikoshi. 2008. Cell proliferation at 122 C and isotopically heavy CH4 production by a hyperthermophilic methanogen under high-pressure cultivation. Proceedings of the National Academy of Sciences 105:10949–10954.

Talbot, P., J.-M. Thébault, A. Dauta, and J. De la Noüe. 1991. A comparative study and mathematical modeling of temperature, light and growth of three microalgae potentially useful for wastewater treatment. Water research 25:465–472.

TALLING, J. F. 1957. PHOTOSYNTHETIC CHARACTERISTICS OF SOME FRESHWATER PLANKTON DIATOMS IN RELATION TO UNDERWATER RADIATION. New Phytologist 56:29–50.

Tan, K., G. Zhou, and S. Ren. 2013. Response of leaf dark respiration of winter wheat to changes in CO2 concentration and temperature. Chinese Science Bulletin 58:1795–1800.

Tang, E. P., R. Tremblay, and W. F. Vincent. 1997. CYANOBACTERIAL DOMINANCE OF POLAR FRESHWATER ECOSYSTEMS: ARE HIGH-LATITUDE MAT-FORMERS ADAPTED TO LOW TEMPERATURE? Journal of Phycology 33:171–181.

Tasaka, Y., Z. Gombos, Y. Nishiyama, P. Mohanty, T. Ohba, K. Ohki, and N. Murata. 1996. Targeted mutagenesis of acyl-lipid desaturases in Synechocystis: evidence for the important roles of polyunsaturated membrane lipids in growth, respiration and photosynthesis. The EMBO journal 15:6416.

Taylor, D., and J. Collie. 2003. Effect of temperature on the functional response and foraging behavior of the sand shrimp Crangon septemspinosa preying on juvenile winter flounder Pseudopleuronectes americanus. Marine Ecology Progress Series 263:217–234.

Temple, G. K., and I. Johnston. 1998. Testing hypotheses concerning the phenotypic plasticity of escape performance in fish of the family Cottidae. The Journal of experimental biology 201:317–331.

Teoh, M.-L., W.-L. Chu, H. Marchant, and S.-M. Phang. 2004. Influence of culture temperature on the growth, biochemical composition and fatty acid profiles of six Antarctic microalgae. J Appl Phycol 16:421–430.

Teoh, M.-L., S.-M. Phang, and W.-L. Chu. 2013. Response of Antarctic, temperate, and tropical microalgae to temperature stress. Journal of applied phycology 25:285–297.

Terrados, J., and J. Ros. 1992. The influence of temperature on seasonal variation of Caulerpa prolifera (Forsskal) Lamouroux photosynthesis and respiration. Journal of Experimental Marine Biology and Ecology 162:199–212.

———. 1995. Temperature effects on photosynthesis and depth distribution of the seagrass Cymodocea nodosa (Ucria) Ascherson in a Mediterranean coastal lagoon: the Mar Menor (SE Spain). Marine Ecology 16:133–144.

Thebud, R., and K. A. Santarius. 1982. Effects of high-temperature stress on various biomembranes of leaf cells in situ and in vitro. Plant physiology 70:200–205.

Thomas, W. H. 1966. EFFECTS OF TEMPERATURE AND ILLUMINANCE ON CELL DIVISION RATES OF THREE SPECIES OF TROPICAL OCEANIC PHYTOPLANKTON. Journal of Phycology 2:17–22.

Thompson, D. J. 1978. Towards a Realistic Predator-Prey Model: The Effect of Temperature on the Functional Response and Life History of Larvae of the Damselfly, Ischnura elegans. The Journal of Animal Ecology 47:757.

Thompson, P. A., M. xin Guo, and P. J. Harrison. 1992. EFFECTS OF VARIATION IN TEMPERATURE. I. ON THE BIOCHEMICAL COMPOSITION OF EIGHT SPECIES OF MARINE PHYTOPLANKTON1. Journal of Phycology 28:481–488.

Throndsen, J. 1976. Occurrence and productivity of small marine flagellates. Norwegian Journal of Botany .

Titus, J. E., and M. S. Adams. 1979. Coexistence and the comparative light relations of the submersed macrophytes Myriophyllum spicatum L. and Vallisneria americana Michx. Oecologia 40:273–286.

Tjoelker, M., J. Oleksyn, and P. Reich. 1998. Seedlings of five boreal tree species differ in acclimation of net photosynthesis to elevated CO2 and temperature. Tree physiology 18:715–726.

Tjoelker, M. G., J. Oleksyn, G. Lorenc-Plucinska, and P. B. Reich. 2009. Acclimation of respiratory temperature responses in northern and southern populations of Pinus banksiana . New Phytologist 181:218–229.

Tjoelker, M. G., J. Oleksyn, P. B. Reich, and R. ŻYTKOWIAK. 2008. Coupling of respiration, nitrogen, and sugars underlies convergent temperature acclimation in Pinus banksiana across wide-ranging sites and populations. Global Change Biology 14:782–797.

Toddl, G. W. 1982. Photosynthesis and respiration of vegetative and reproductive parts of wheat and barley plants in response to increasing temperature. Pages 57–62 *in* Proceedings of the Oklahoma Academy of Science. Vol. 62.

Tomas, C. R. 1978. OLISTHODISCUS LUTEUS (CHRYSOPHYCEAE) I. EFFECTS OF SALINITY AND TEMPERATURE ON GROWTH. MOTILITY AND SURVIVALS2. Journal of Phycology 14:309–313.

Torres-Contreras, H., and R. Vasquez. 2004. A field experiment on the influence of load transportation and patch distance on the locomotion velocity of Dorymyrmex goetschi (Hymenoptera, Formicidae). Insectes Sociaux 51:265–270.

Torzillo, G., and A. Vonshak. 1994. Effect of light and temperature on the photosynthetic activity of the cyanobacterium Spirulina platensis. Biomass and Bioenergy 6:399–403.

Traniello, J. F. A., M. S. Fujita, and R. V. Bowen. 1984. Ant foraging behavior: ambient temperature influences prey selection. Behavioral Ecology and Sociobiology 15:65–68.

Tranquillini, W., W. M. Havranek, and P. Ecker. 1986. Effects of atmospheric humidity and acclimation temperature on the temperature response of photosynthesis in young Larix decidua Mill. Tree Physiology 1:37–45.

Tsujimura, S., K. Ishikawa, and H. Tsukada. 2001. Effect of temperature on growth of the cyanobacterium Aphanizomenon flos-aquae in Lake Biwa and Lake Yogo. Phycological Research 49:275–280.

Tsujimura, S., M. Kumagai, J. Urabe, T. Sekino, Y. Hayami, and M. Maruo. 2003. Effect of temperature and light on growth of planktic green algae isolated from Lake Hövsgöl, Mongolia. Algological Studies 110:81–89.

Tsujimura, S., and T. Okubo. 2003. Development of Anabaena blooms in a small reservoir with dense sediment akinete population, with special reference to temperature and irradiance. Journal of Plankton Research 25:1059–1067.

Tyler, A. V. 1970. Rates of Gastric Emptying in Young Cod. J. Fish. Res. Bd. Can. 27:1177–1189.

Ueda, Y., S. Nishihara, H. Tomita, and Y. Oda. 2000. Photosynthetic response of Japanese rose species Rosa bracteata and Rosa rugosa to temperature and light. Scientia Horticulturae 84:365–371.

Uehlinger, U. 1981. Experimentelle Untersuchungen zur Autökologie von Aphanizomenon flos-aquae. Algological Studies/Archiv für Hydrobiologie, Supplement Volumes pages 260–288.

Usup, G., D. M. Kulis, and D. M. Anderson. 1994. Growth and toxin production of the toxic dinoflagellatePyrodinium bahamense var.compressum in laboratory cultures. Nat. Toxins 2:254–262.

Valík, Ľ., A. Medveďová, M. Čižniar, and D. Liptáková. 2013. Evaluation of temperature effect on growth rate of Lactobacillus rhamnosus GG in milk using secondary models. Chemical Papers 67.

Van Auken, O. W., and I. B. McNULTY. 1973. The effect of environmental factors on the growth of a halophylic species of algae. The Biological Bulletin 145:210–222.

Van der Westhuizen, A., and J. Eloff. 1985. Effect of temperature and light on the toxicity and growth of the blue-green alga Microcystis aeruginosa (UV-006). Planta 163:55–59.

van Liere, E. 1979. On Oscillatoria agardhii Gomont: experimental ecology and physiology of a nuisance bloomforming cyanobacterium. " De Nieuwe Schouw" Press.

Vapaavuori, E., R. Rikala, and A. Ryyppö. 1992. Effects of root temperature on growth and photosynthesis in conifer seedlings during shoot elongation. Tree Physiology 10:217–230.

Venter, A., A. Jordaan, and A. Pieterse. 2003. Oscillatoria simplicissima: a taxonomical study. WATER SA-PRETORIA- 29:101–104.

Vona, V., V. D. M. Rigano, O. Lobosco, S. Carfagna, S. Esposito, and C. Rigano. 2004. Temperature responses of growth, photosynthesis, respiration and NADH: nitrate reductase in cryophilic and mesophilic algae. New Phytologist 163:325–331.

Vose, J. M., and P. V. Bolstad. 1999. Challenges to modelling NPP in diverse eastern deciduous forests: species-level comparisons of foliar respiration responses to temperature and nitrogen. Ecological Modelling 122:165–174.

Wakeling, J. M., N. J. Cole, K. M. Kemp, and I. A. Johnston. 2000. The biomechanics and evolutionary significance of thermal acclimation in the common carp Cyprinus carpio. American Journal of Physiology-Regulatory, Integrative and Comparative Physiology 279:R657–R665.

Walcroft, A., X. L. Roux, A. Diaz-Espejo, N. Dones, and H. Sinoquet. 2002. Effects of crown development on leaf irradiance, leaf morphology and photosynthetic capacity in a peach tree. Tree Physiology 22:929–938.

Waldschmidt, S., and C. R. Tracy. 1983. Interactions between a Lizard and Its Thermal Environment: Implications for Sprint Performance and Space Utilization in the Lizard Uta Stansburiana. Ecology 64:476–484.

Wallace, J. 1973*a*. Observations on the relationship between the food consumption and metabolic rate of Blennius pholis L. Comparative Biochemistry and Physiology Part A: Physiology 45:293–306.

Wallace, J. C. 1973*b*. Feeding, starvation and metabolic rate in the shore crab Carcinus maenas. Marine Biology 20:277–281.

Walne, P. R., and G. J. Dean. 1972. Experiments on Predation by the Shore Crab, Carcinus Maenas L., on Mytilus and Mercenaria. ICES Journal of Marine Science 34:190–199.

Wang, K.-Y., S. Kellomäki, and K. Laitinen. 1996. Acclimation of photosynthetic parameters in Scots pine after three years exposure to elevated temperature and CO 2. Agricultural and Forest Meteorology 82:195–217.

Wang, X., K. Tang, Y. Wang, and W. Smith. 2010. Temperature effects on growth, colony development and carbon partitioning in three Phaeocystis species. Aquat. Biol. 9:239–249.

WARD, D. A., and D. W. LAWLOR. 1990. Abscisic acid may mediate the rapid thermal acclimatization of photosynthesis in wheat. Journal of experimental botany 41:309–314.

Ware, D. M. 1973. Risk of Epibenthic Prey to Predation by Rainbow Trout (Salmo gairdneri) . J. Fish. Res. Bd. Can. 30:787–797.

Warren, C. 2006. Temperature response of photosynthesis and internal conductance to CO2: results from two independent approaches. Journal of Experimental Botany 57:3057–3067.

———. 2008. Does growth temperature affect the temperature responses of photosynthesis and internal conductance to CO2? A test with Eucalyptus regnans. Tree Physiology 28:11–19.

Watras, C. J., S. W. Chisholm, and D. M. Anderson. 1982. Regulation of growth in an estuarine clone of Gonyaulax tam arensis Lebour: Salinity-dependent temperature responses. Journal of Experimental Marine Biology and Ecology 62:25–37.

Weatherhead, P. J., and I. C. Robertson. 1992. Thermal constraints on swimming performance and escape response of northern water snakes (Nerodia sipedon) . Can. J. Zool. 70:94–98.

Wedge, R. M., and J. E. Burris. 1982. Effects of light and temperature on duckweed photosynthesis. Aquatic Botany 13:133–140.

Weier, J. A., and D. H. Feener. 1995. Foraging in the seed-harvester ant genus Pogonomyrmex: are energy costs important? Behavioral Ecology and Sociobiology 36:291–300.

Weiler, C., and R. Eppley. 1979. Temporal pattern of division in the dinoflagellate genus Ceratium and its application to the determination of growth rate. Journal of Experimental Marine Biology and Ecology 39:1–24.

Weis, E. 1981. Reversible heat-inactivation of the calvin cycle: A possible mechanism of the temperature regulation of photosynthesis. Planta 151:33–39.

Whitehead, P., J. Puckridge, C. Leigh, and R. Seymour. 1989. Effect of temperature on jump performance of the frog Limnodynastes tasmaniensis. Physiological zoology pages 937–949.

Widdows, J. 1973. Effect of temperature and food on the heart beat, ventilation rate and oxygen uptake of Mytilus edulis. Marine Biology 20:269–276.

Wilken, S., J. Huisman, S. Naus-Wiezer, and E. Donk. 2013. Mixotrophic organisms become more heterotrophic with rising temperature. Ecology letters 16:225–233.

William, K. L., and I. Morris. 1982. Temperature adaptation in Phaeodactylum tricornutum Bohlin: photosynthetic rate compensation and capacity. Journal of Experimental Marine Biology and Ecology 58:135–150.

WISE, R. R., A. J. OLSON, S. M. SCHRADER, and T. D. SHARKEY. 2004. Electron transport is the functional limitation of photosynthesis in field-grown Pima cotton plants at high temperature. Plant, Cell and Environment 27:717–724.

Wittmann, C., and H. Pfanz. 2007. Temperature dependency of bark photosynthesis in beech (Fagus sylvatica L.) and birch (Betula pendula Roth.) trees. Journal of Experimental Botany 58:4293–4306.

Wootton, R. J., J. R. M. Allen, and S. J. Cole. 1980. Effect of body weight and temperature on the maximum daily food consumption of Gasterosteus aculeatus L. and Phoxinus phoxinus (L.): selecting an appropriate model. Journal of Fish Biology 17:695–705.

Xiong, F. S., E. C. Mueller, and T. A. Day. 2000. Photosynthetic and respiratory acclimation and growth response of Antarctic vascular plants to contrasting temperature regimes. American Journal of Botany 87:700–710.

Xu, N., S. Duan, A. Li, C. Zhang, Z. Cai, and Z. Hu. 2010. Effects of temperature, salinity and irradiance on the growth of the harmful dinoflagellate Prorocentrum donghaiense Lu. Harmful Algae 9:13–17.

Xue, Y., P. Zhou, Y. Ma, Y. Liu, and Y. Xu. 2001. Thermoanaerobacter tengcongensis sp. nov., a novel anaerobic, saccharolytic, thermophilic bacterium isolated from a hot spring in Tengcong, China. International Journal of Systematic and Evolutionary Microbiology 51:1335–1341.

Yamaguchi, H., K. Mizushima, S. Sakamoto, and M. Yamaguchi. 2010. Effects of temperature, salinity and irradiance on growth of the novel red tide flagellate Chattonella ovata (Raphidophyceae). Harmful Algae 9:398–401.

Yamaguchi, H., T. Yoshimatsu, Y. Tanimoto, S. Sato, T. Nishimura, K. Uehara, and M. Adachi. 2012. Effects of temperature, salinity and their interaction on growth of the benthic dinoflagellate Ostreopsis cf. ovata (Dinophyceae) from Japanese coastal waters. Phycological research 60:297–304.

Yamaguchi, M., and T. Honjo. 1989. . NIPPON SUISAN GAKKAISHI 55:2029–2036.

Yamaguchi, M., I. Shigeru, K. Nagasaki, Y. Matsuyama, T. Uchida, and I. Imai. 1997. Effects of temperature and salinity on the growth of the red tide flagellates Heterocapsa circularisquama (Dinophyceae) and Chattonella verruculosa (Raphidophyceae). Journal of Plankton Research 19:1167–1174.

Yamamoto, A. C., A. K. Doetzer, and L. A. Foerster. 2005. Efeito da temperatura no desenvolvimento de Euplectrus ronnai Brèthes (Hymenoptera, Eulophidae) parasitando lagartas de Pseudaletia sequax Fraquelemont (Lepidoptera, Noctuidae) e consumo alimentar do hospedeiro. Acta Biológica Paranaense 27.

Yamamoto, Y., and H. Nakahara. 2005. The formation and degradation of cyanobacterium Aphanizomenon flos-aquae blooms: the importance of pH, water temperature, and day length. Limnology 6:1–6.

Yamasaki, T., T. Yamakawa, Y. Yamane, H. Koike, K. Satoh, and S. Katoh. 2002. Temperature Acclimation of Photosynthesis and Related Changes in Photosystem II electron transport in winter wheat. PLANT PHYSIOLOGY 128:1087–1097.

Yamatogi, T., M. Sakaguti, N. Takagi, M. Iwataki, and K. Matsuoka. 2005. Effects of temperature, salinity and light intensity on the growth of a harmful dinoflagellate Cochlodinium polykrikoides Margalef occurring in coastal waters of West Kyushu, Japan. Bulletin of the Plankton Society of Japan (Japan) .

Yamori, W., K. Noguchi, and I. Terashima. 2005. Temperature acclimation of photosynthesis in spinach leaves: analyses of photosynthetic components and temperature dependencies of photosynthetic partial reactions. Plant, Cell & Environment 28:536–547.

YANASE, R., and T. Imai. 1968. The effect of light intensity and temperature on the growth of several marine algae useful for rearing molluscan larvae. Tohoku journal of agricultural research 19:75–82.

Yang, X., Z. Liang, and C. Lu. 2005. Genetic engineering of the biosynthesis of glycinebetaine enhances photosynthesis against high temperature stress in transgenic tobacco plants. Plant Physiology 138:2299–2309.

Yoder, J. A. 1979. EFFECT OF TEMPERATURE ON LIGHT-LIMITED GROWTH AND CHEMICAL COMPOSITION OF SKELETONEMA COSTATUM (BACILLARIOPHYCEAE). Journal of Phycology 15:362–370.

Zamani, A., A. Talebi, Y. Fathipour, and V. Baniameri. 2006. Temperature-dependent functional response of two aphid parasitoids, Aphidius colemani and Aphidius matricariae (Hymenoptera: Aphidiidae), on the cotton aphid. J Pest Sci 79:183–188.

Zargar, S., and T. Ghosh. 2007. Thermal and biocidal (chlorine) effects on select freshwater plankton. Archives of environmental contamination and toxicology 53:191–197.

Zargar, S., K. Krishnamurthi, S. S. Devi, T. Ghosh, and T. Chakrabarti. 2006. Temperature-induced stress on growth and expression of Hsp in freshwater alga Scenedesmus quadricauda. Biomedical and Environmental Sciences 19:414.

Zehnder, A. J. B., and K. Wuhrmann. 1977. Physiology of a Methanobacterium strain AZ. Archives of Microbiology 111:199–205.

Zeikus, J., and R. Wolee. 1972. Methanobacterium thermoautotrophicus sp. n., an anaerobic, autotrophic, extreme thermophile. Journal of Bacteriology 109:707–713.

Zhang, B.-Y., Y.-G. Li, Z.-K. Li, Y.-H. Geng, and H.-J. Hu. 2003. Effects of temperature, light intensity and pH on photosynthesis and growth rate of Haematococcus pluvialis. Oceanologia Et Limnologia Sinica 34:558–565.

ZIMMERMAN, M. C., and T. E. WISSING. 1978. Effects of temperature on gut-loading and gut-clearing times of the burrowing mayfly, Hexagenia limbata. Freshwater Biology 8:269–277.

Zimmerman, R. C., R. D. Smith, and R. S. Alberte. 1989. Thermal acclimation and whole-plant carbon balance in Zostera marina L.(eelgrass). Journal of Experimental Marine Biology and Ecology 130:93–109.

Zinder, S., T. Anguish, and S. Cardwell. 1984. Effects of temperature on methanogenesis in a thermophilic (58 C) anaerobic digestor. Applied and Environmental Microbiology 47:808–813.

Zinder, S. H., and R. A. Mah. 1979. Isolation and characterization of a thermophilic strain of Methanosarcina unable to use H2-CO2 for methanogenesis. Applied and Environmental Microbiology 38:996–1008.

Zinser, E. R., Z. I. Johnson, A. Coe, E. Karaca, D. Veneziano, and S. W. Chisholm. 2007. Influence of light and temperature on Prochlorococcus ecotype distributions in the Atlantic Ocean. Limnol. Oceangr. 52:2205–2220.

Ziska, L. H. 2001. Growth temperature can alter the temperature dependent stimulation of photosynthesis by elevated carbon dioxide in Albutilon theophrasti. Physiol Plant 111:322–328.

Zlotnik, I., and Z. Dubinsky. 1989. The effect of light and temperature on DOC excretion by phytoplankton. Limnology and Oceanography 34:831–839.
